# Comparison of eight complete chloroplast genomes of the endangered *Aquilaria* tree species (Thymelaeaceae) and their phylogenetic relationships

**DOI:** 10.1038/s41598-020-70030-0

**Published:** 2020-08-03

**Authors:** Muhammad Syahmi Hishamuddin, Shiou Yih Lee, Wei Lun Ng, Shairul Izan Ramlee, Dhilia Udie Lamasudin, Rozi Mohamed

**Affiliations:** 1 Forest Biotechnology Laboratory, Department of Forestry Science and Biodiversity, Faculty of Forestry and Environment, Universiti Putra Malaysia, 43400 Serdang, Selangor, Malaysia; 2 China-ASEAN College of Marine Sciences, Xiamen University Malaysia, 43900 Sepang, Selangor, Malaysia; 3 Department of Crop Science, Faculty of Agriculture, Universiti Putra Malaysia, 43400 Serdang, Selangor, Malaysia; 4 Department of Cell and Molecular Biology, Faculty of Biotechnology and Biomolecular Sciences, Universiti Putra Malaysia, 43400 Serdang, Selangor, Malaysia; 5 Halal Products Research Institute, Universiti Putra Malaysia, 43400 Serdang, Selangor, Malaysia

**Keywords:** Biotechnology, Genetics, Plant sciences

## Abstract

*Aquilaria* tree species are naturally distributed in the Indomalesian region and are protected against over-exploitation. They produce a fragrant non-timber product of high economic value, agarwood. Ambiguous species delimitation and limited genetic information within *Aquilaria* are among the impediments to conservation efforts. In this study, we conducted comparative analysis on eight *Aquilaria* species complete chloroplast (cp) genomes, of which seven were newly sequenced using Illumina HiSeq X Ten platform followed by de novo assembly. *Aquilaria* cp genomes possess a typical quadripartite structure including gene order and genomic structure. The length of each of the cp genome is about 174 kbp and encoded between 89 and 92 proteins, 38 tRNAs, and 8 rRNAs, with 27 duplicated in the IR (inverted repeat) region. Besides, 832 repeats (forward, reverse, palindrome and complement repeats) and nine highly variable regions were also identified. The phylogenetic analysis suggests that the topology structure of *Aquilaria* cp genomes were well presented with strong support values based on the cp genomes data set and matches their geographic distribution pattern. In summary, the complete cp genomes will facilitate development of species-specific molecular tools to discriminate *Aquilaria* species and resolve the evolutionary relationships of members of the Thymelaeaceae family.

## Introduction

*Aquilaria* Lam., is a tropical tree genus from the family Thymelaeaceae. Members of this taxon are widely distributed in the Indomalesia region, but their numbers are declining in the wild. Consequentially, they have entered various Red List categories of the International Union for Conservation of Nature (IUCN). The latest IUCN publication placed four species (*A. crassna*, *A. khasiana*, *A. malaccensis*, and *A. rostrata*) under “Critically Endangered”; one species (*A. microcarpa*) as “Endangered”; nine species (*A. banaensis*, *A. beccariana*, *A. cumingiana*, *A. decemcostata*, *A. filaria*, *A. hirta*, *A. rugosa*, *A. sinensis*, *A. yunnanensis*) as “Vulnerable”; and seven species (*A. apiculata*, *A. baillonii*, *A. brachyantha*, *A. citrinicarpa*, *A. parvifolia*, *A. subintegra*, *A. urdanetensis*) as “Data Deficient” (IUCN, www.iucnredlist.org, accessed on 6 November 2019). *Aquilaria* produces a fragrant resin (agarwood) as a self-healing mechanism to external wounding of its trunk, branches and roots^1^. Agarwood is a valuable raw material in production of perfumes, incense and traditional medicines^2^. The high demand in agarwood has led to illegal logging and non-selective felling due to the low percentage of naturally occurring agarwood trees in the wild^2,3^. The Convention on International Trade in Endangered Species of Wild Fauna and Flora (CITES) has listed all *Aquilaria* species under Appendix II, as one of the countermeasures to reduce illegal agarwood trade^2,4^.

Agarwood is chiefly traded in wood form or consumer products, yet *Aquilaria* species delimitation is based on the plant’s botanical characteristics, with much emphasis given to the reproductive parts specifically flower and fruit^5^. Incomplete distinct morphological characteristics render genetic detection tools indispensable during agarwood identification process. Recent approaches have utilized short DNA sequences to identify *Aquilaria* species and species-origin of agarwood^6–9^. Short cp gene sequences have been adequate in barcoding some land plants^10^, unfortunately it has been shown that with *Aquilaria*, highly evolved DNA barcodes are required. Several DNA barcoding loci have been tested in *Aquilaria*, yet the discrimination power across a majority of the *Aquilaria* species is insufficient^7^.

A recent phylogenetic analysis utilizing five non-coding cp DNA regions from 15 species (Aquilarieae) yielded inconclusive resolution due to the high percentage of conserved sites^11^. Meanwhile, in the same study, nuclear ribosomal DNA internal transcribed spacer (ITS) revealed a paraphyletic relationship between *Aquilaria* species from Indochina and Malesian. It has been suggested that genome-scale data could provide a better resolution and thorough information of a studied genus pertaining to taxonomical aspects, genetic diversity, and pattern of evolution^11^.

Chloroplast is an important organelle contributing to the growth development of most plants. They play important roles in photosynthesis and carbon fixation^12^. Chloroplast or plastid originally derived from a free-living photosynthetic prokaryote. Present-day chloroplast arose from a cynobacterial endosymbiont^13^. Chloroplasts still exhibit many prokaryotic characteristics such as having a circular DNA, reproducing in a similar way as bacteria through division, and importing nuclear encoded proteins through thylakoids^14^. In general, the cp genomes in angiosperms are circular DNA molecules with a highly conserved region, gene content, and gene order, and the standard cp genome size is ranged between 120 and 170 kbp in length^15^. A typical cp genome consists of a pair of inverted repeats (IR) region that is separated by a large single copy (LSC) region and a small single copy (SSC) region^16^. The advancement of high-throughput sequencing technology has amassed thousands of complete cp genomes from various land plants in just several years^17^. A total of 23,867 complete cp genomes of land plants have been sequenced (NCBI, https://www.ncbi.nlm.nih.gov/genome/organelle/, accessed on 12 February 2020). The maternal inheritance in the cp genome has provided an exclusive and substantial information for plant systematics and evolutionary relationships^18^. Cp genomes have been used in species identification, phylogeny and population genetic analyses^17,19,20^. Potential markers can be developed through cp genomes analysis for identification of plant species, particularly for the taxonomically complicated groups^10,21^.

Due to the small genome size, high interspecific and low intraspecific divergence, and ease of handling, cp genome is an attractive alternative to provide more variations in discriminating closely related plants^10^. To date, research on *Aquilaria* genomics has yielded a draft whole genome of *A. agallocha*^22^ and complete sequences of six cp genomes, one each from *A. crassna*^23^, *A. malaccensis*^24^, and *A. yunnanensis*^25^, and three from *A. sinensis*^18,26,27^. In this study, we report complete cp genome sequences of seven *Aquilaria* species and incorporate these new sequences together with another published cp genome from our group (*A. malaccensis*)^24^ in all our analyses. In addition, we also completely sequenced the cp genomes of *Gonystylus affinis* and *Phaleria macrocarpa* and retrieved complete cp sequences of another six species, all of which are under the Thymelaeaceae family, to determine their molecular placement within the phylogenetic tree. Collectively, we provide a rich genomic resource to better understand *Aquilaria*, which may help facilitate the conservation efforts of these endangered species.

## Materials and methods

### Sample materials

Fresh leaf samples were collected from individual trees growing in the greenhouse of the Faculty of Forestry and Environment, Universiti Putra Malaysia (UPM), Serdang, Selangor, Malaysia, and the *Aquilaria* germplasm of Forest Research Institute of Malaysia (FRIM), Kepong, Selangor, Malaysia. For comparative analysis, seven species, *A. beccariana*, *A. hirta*, *A. microcarpa*, *A. rostrata*, *A. crassna*, *A. sinensis*, and *A. subintegra*, were sequenced. The four former species are native to Malaysia, while the following three are introduced plantation species in the country. For phylogenetic analysis, the cp genome of *G. affinis* and *P. macrocarpa*, two close relatives of *Aquilaria*, were also sequenced.

### DNA extraction and sequencing

A total of 100 mg fresh leaves was pulverized into powder using mortar and pestle, with the aid of liquid nitrogen. Total genomic DNA was extracted using a modified cetyltrimethylammonium bromide (CTAB) method^28^. The quantity and quality of the DNA samples were determined using the Qubit dsDNA BR assay (Life Technologies, Carlsbad, CA, USA) using the manufacturer's instructions. DNA samples were fragmented using sonication, purified and end-repaired, and their sizes were determined by gel electrophoresis and the size of fragments were between 200 to 500 bp. A genomic library with an insert size of 300 bp was prepared using TruSeq DNA Sample Prep Kit (Illumina, CA, USA) and next-generation sequencing was conducted on a HiSeq X Ten platform (Illumina, USA).

### Chloroplast genome assembly and annotation

Approximately 8 Gb of raw data that consisted of 150-bp paired-end reads were generated and the sequence adaptors for the raw reads were trimmed off using the base quality control software NGS QC Toolkit v2.3.3^29^. The cp genome was assembled using NOVOPlasty v3.8.2^30^, with the *rbc*L sequence of *A. yunnanensis* (KR528756) as the seed sequence. The assembled cp genome sequence was annotated using online annotation tool GeSeq^31^, and further compared manually against *A. yunnanensis* cp genome (MG656407). The circular cp genome maps were visualized using OGDRAW v1.3.1^32^.

### Comparative analysis of *Aquilaria* chloroplast genomes

For comparative cp genome analysis, the sequence of *A. malaccensis* (MH286934) was included. Base composition and GC content were determined using DNA Baser Sequence Assemble v5.15 (https://www.dnabaser.com/) and Emboss (https://www.bioinformatics.nl/cgi-bin/emboss/geecee), respectively. Sequences were aligned using MAFFT v7^33^ with default settings (strategy of FFT-NS-2) and then transferred into DnaSP v5.10.1^34^ to identify nucleotide diversity in the total genome, LSC, SSC and IR regions. The boundaries between the IR and SC regions were further evaluated manually to examine the differences in length variation in the cp genomes of *Aquilaria*.

### Repeat structure analysis and identification of highly variable regions

Repeat sequences as well as forward (F), reverse (R), complement (C) and palindrome (P) sequences were identified using REPuter^35^, with the maximum and minimum repeat size set at 50 and 30, respectively, and Hamming distance ≤ 3. To identify highly variable regions, polymorphic sites and nucleotide variability (Pi) in the eight MAFFT aligned cp genomes were evaluated using a sliding window analysis available in DnaSP v5.10.01, under a 200-bp step size and a 600-bp window length. The regions that contain the number of polymorphic sites that are more than the sum of the average and double the standard deviation are regarded as highly variable regions in the cp genome^34^.

### Phylogenetic analysis

Phylogenetic analysis was performed to determine relatedness of the seven *Aquilaria* cp genomes sequenced in this study and four publicly available *Aquilaria* cp genomes: (1) *A. crassna* (MK779998)^23^, (2) *A. sinensis* (KT148967)^18^, (3) *A. yunnanensis* (MG656407)^25^, and (4) *A. malaccensis* (MH286934)^24^. To place *Aquilaria* in relation to other species in the family Thymelaeaceae, sequences from *G. affinis* and *P. macrocarpa*, and three available accessions: (1) *Daphne tangutica* (MK455880)^36^, (2) *Daphne kiusiana* (KY991380)^37^, and (3) *Stellera chamaejasme* (MK681211)^38^, were included. *Neobalanocarpus heimii* (MH746730)^39^ and *Eucalyptus grandis* (HM347959)^40^ were used as outgroups. Sequences were aligned using MAFFT v7^33^ with default settings (strategy of FFT-NS-2). Phylogenetic analyses were subsequently performed using Maximum likelihood (ML) and Bayesian inference (BI) methods. Maximum likelihood (ML) analyses were performed using IQ-TREE v.1.4.2^41^ with branch support estimated using 2,000 replicates of both SH-like approximate likelihood-ratio test (SH-aLRT)^42^ and the ultrafast bootstrapping algorithm (UFboot)^43^. The ModelFinder option was used to identify the optimal partitioning scheme and substitution models^44^, in which the DNA substitution model that is most suitable for our dataset was transversion model (TVM) with empirical base frequencies (+ F) and discrete Gamma model with default 4 rate categories (+ G4) (= TVM + F + G4). The phylogenetic tree was rooted using *E. grandis* and visualized using Figtree v1.4.4^45^. Bayesian inference (BI) analyses were performed using the program MrBayes v3.2.7^46^. Markov chain Monte Carlo (MCMC) simulations were run twice independently for 2 million generations, and sampling trees every 100 generations. Convergence was determined by examining the average standard deviation of split frequencies (≤ 0.01). The first 25% of trees was discarded as burn-in, and the remaining trees were used to build a majority-rule consensus tree.

## Results and discussion

### Chloroplast genome sequencing

Approximately 60,000,000 raw reads were obtained for each species sequenced using the HiSeq X Ten system. Raw reads were inserted directly into the pipeline without filtering or quality trimming to obtain maximum useful data. To accelerate the assembly of plastid genomes, we selected only the first 13.6 million sequences of each paired-end data, yielding with a total of 15.4 Gb clean data after sequencing. For base quality assessment, 94.7% of Q30 bases were obtained. All newly sequenced cp genomes of *Aquilaria* species with gene annotations have been deposited into the GenBank (*A. beccariana*, MN125347; *A. crassna*, MN125348; *A. hirta,* MN125349; *A. microcarpa,* MN125350; *A. rostrata*, MN125351; *A. sinensis,* MN147870; *A. subintegra*, MN147871). In addition, we also sequenced cp genomes of selected species of the two closely related taxa, *G. affinis* (MN147872) and *P. macrocarpa* (MN147873), due to the limited number of available cp genome sequences for Thymelaeaceae.

### Complete chloroplast genomes of *Aquilaria* species

**Figure 1:**
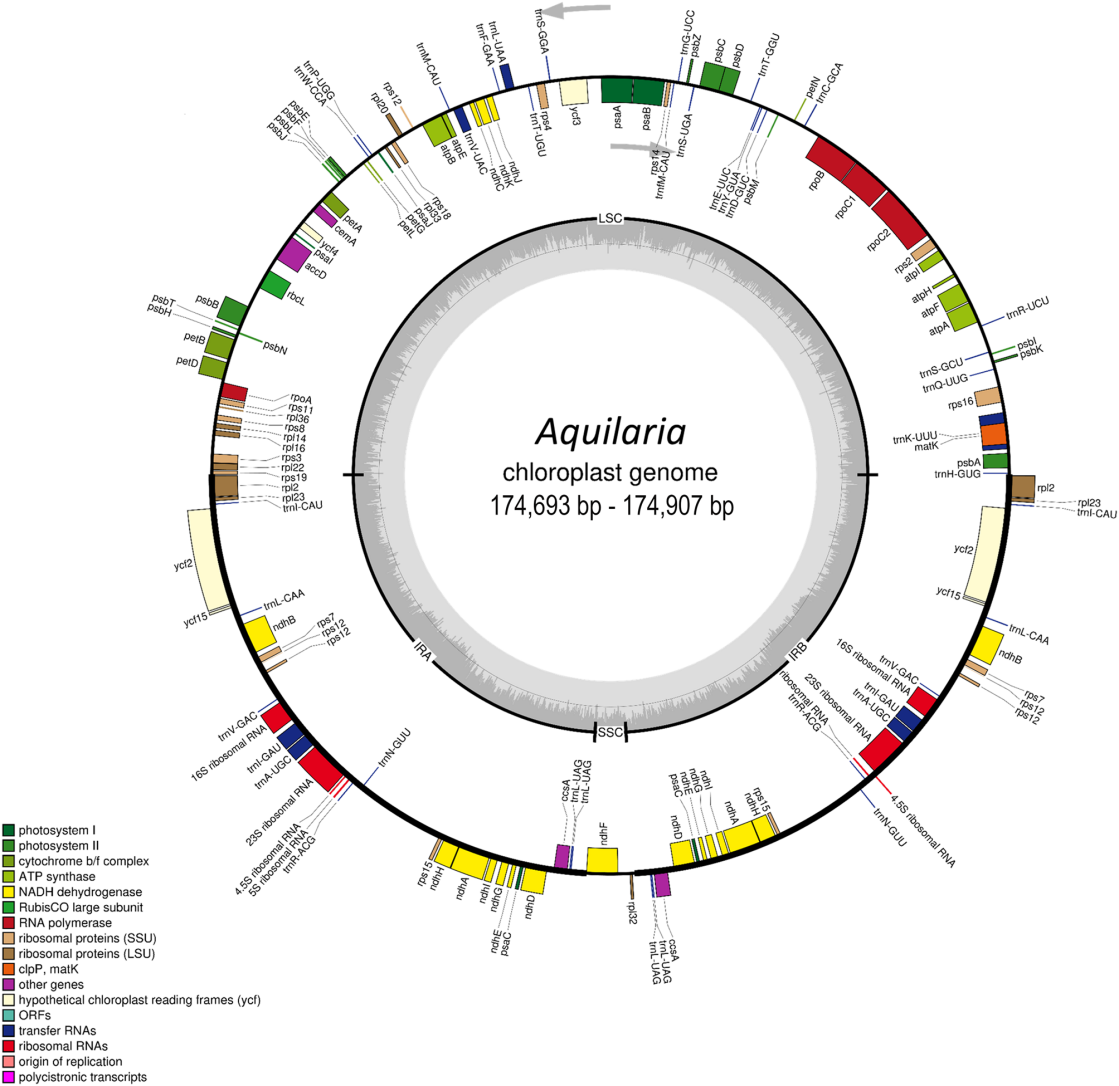
Chloroplast genome maps of eight *Aquilaria* species (*A. beccariana*, *A. crassna*, *A. hirta*, *A. malaccensis*, *A. microcarpa*, *A. rostrata*, *A. sinensis* and *A. subintegra*). Genes inside outer ring are transcribed clockwise, while genes outside outer ring are transcribed counterclockwise. Genes of different functional groups are shown in colored bars. The inner circle (dashed gray area) indicates the proportional GC content of the corresponding genes. Regions of the large single-copy (LSC), small single-copy (SSC) and inverted repeats (IR_A_ and IR_B_) are indicated.

The cp genomes of the *Aquilaria* species are different in size by only 68–214 bp, from the smallest, 174,693 bp (*A. rostrata*) to the biggest 174,907 bp (*A. sinensis*) (Table 1). All eight *Aquilaria* cp genomes share a typical quadripartite structure composed of a pair of IRs known as IR_A_ and IR_B_, and a single LSC and SSC (Fig. 1). In addition, the gene content and order are highly similar. This agrees with the consensus that the genomic structure in cp genomes of angiosperms is highly conserved^15,17^. The length of LSC ranged from 87,221 bp (*A.hirta)* to 87,355 bp (*A. sinensis*), while the length of SSC and IR ranged from 3,233 bp (*A. rostrata*) to 3,347 bp (*A. malaccensis*), and from 42,085 bp (*A. microcarpa*) to 42,102 bp (*A. sinensis*), respectively. The GC content is highest in IR (~ 38%), moderate in LSC (~ 34%) and lowest in SSC (~ 29%) (Table 1). Furthermore, the GC contents of LSC and SSC in all *Aquilaria* species are much lower than IR (Table 1) because of the reduction of AT nucleotides in the five rRNA genes (*rrn23s*, *rrn16s*, *rrn12s*, *rrn4.5 s*, and *rrn5s*)^47,48^ (Table 2). The GC content of the *Aquilaria* species in our study is similar to that reported in *A. yunnanensis* (38%)^25^ and *A. sinensis* (36.7%)^26^. The contraction and expansion of IR region boundaries are considered the primary mechanism that affects the varying lengths in angiosperm cp genomes, as demonstrated in Apiales^49^ and Trochodendraceae^50^. However, in this study, variations were in fact detected at the LSC/IR_A_, IR_A_/SSC, SSC/IR_B_, and IR_B_/LSC border regions of the *Aquilaria* cp genomes (Fig. 3). When comparing the boundary (IR/SC) regions between *Aquilaria* species and two of their Thymelaeaceae relatives, *S. chamaejasme* (MK681211) and *D. kiusiana* (KY991380), they all share highly identical genes at the border junctions.

**Table 1:** Summary of the assembly data of eight *Aquilaria* chloroplast genomes.

Species	*Aquilaria beccariana*	*Aquilaria crassna*	*Aquilaria hirta*	*Aquilaria malaccensis*	*Aquilaria microcarpa*	*Aquilaria rostrata*	*Aquilaria sinensis*	*Aquilaria subintegra*
Genome size (bp)	174,831	174,830	174,761	174,832	174,819	174,693	174,907	174,828
LSC length (bp)	87,301	87,281	87,221	87,302	87,298	87,255	87,355	87,279
SSC length (bp)	3,347	3,347	3,343	3,347	3,348	3,233	3,345	3,344
IR length (bp)	42,090	42,090	42,097	42,090	42,085	42,101	42,102	42,101
GC content (%)	36.7	36.7	36.7	36.7	36.7	36.7	36.7	36.7
GC content in LSC (%)	34.9	34.9	34.9	34.9	34.9	35.0	34.9	34.9
GC content in SSC (%)	29.2	29.1	29.4	29.2	29.3	29.5	29.0	29.1
GC content in IR (%)	38.9	38.8	38.9	38.9	38.9	38.9	38.8	38.8
Protein-coding genes	89	89	90	92	90	89	89	89
tRNA genes	38	38	38	38	38	38	38	38
rRNA genes	8	8	8	8	8	8	8	8

Data were extracted from the complete chloroplast genomes sequenced in this study and the available *A. malaccensis* sequences (GenBank accession number MH286934).

**Table 2:** List of annotated genes in *Aquilaria* chloroplast genomes.

Category of Genes	Group of Gene					
Self-replication	Ribosomal RNA genes	*rrn*23s^(x2)^	*rrn*16s^(x2)^	*rrn*12s^(x2)^	*rrn*4.5s^(x2)^	*rrn*5s^(x2)^
	Transfer RNA genes	*trn*A*-*UGC^(x2)^ *trn*fM-CAU *trn*I-GAU^(x2)^ *trn*M-CAU *trn*R-UCU *trn*T-UGU	*trn*C-GCA *trn*G-GCC *trn*K-UUU *trn*N-GUU^(x2)^ *trn*S-GCU *trn*V-GAC^(x2)^	*trn*D-GUC *trn*G-UCC *trn*L-CAA^(x2)^ *trn*P-UGG *trn*S-GGA *trn*V-UAC	*trn*E-UUC *trn*H-GUG *trn*L-UAA *trn*Q-UUG *trn*S-UGA *trn*W-CCA	*trn*F-GAA *trn*I-CAU^(x2)^ *trn*L-UAG^(x2)^ *trn*R-ACG^(x2)^ *trn*T-GGU *trn*Y-GUA
	Small subunit of ribosome	*rps*2 *rps*11 *rps*18^**a**^	*rps*3 *rps*12^(x2)^ *rps*19	*rps*4 *rps*14	*rps*7^(x2)^ *rps*15^(x2)^	*rps*8 *rps*16
	Large subunit of ribosome	*rpl*2^(x2)^ *rpl*23^(x2)^	*rpl*14 *rpl*32	*Rpl*16^**b**^ *rpl*33	*rpl*20 *rpl*36	*rpl*22
	DNA-dependent RNA polymerase	*rpo*A	*rpo*B	*rpo*C1	*rpo*C2	
	Subunit of NADH-dehydrogenase	*ndh*A^(x2)^ *ndh*F *ndhK*	*ndh*B^(x2)^ *ndh*G^(x2)^	*ndh*C *ndh*H^(x2)^	*ndh*D^(x2)^ *ndh*I^(x2)^	*ndh*E^(x2)^ *ndh*J
	Subunits of photosystem I	*psa*A	*psa*B	*psa*J	*psa*I	*psa*C^(x2)^
Genes for Photosynthesis	Subunits of photosystem II	*psb*A *psb*F *psb*L	*psb*B *psb*H *psb*M	*psb*C *psb*I *psb*N	*psb*D *psb*J *psb*T	*psb*E *psb*K *psb*Z
	Subunits of Cytochrome b/f complex	*pet*A	*pet*B	*pet*D	*pet*L	*pet*N
	Subunits of ATP synthase	*atp*A *atp*I	*atp*B	*atp*E	*atp*F	*atp*H
	Subunits of rubisco	*rbc*L				
	Maturase	*mat*K				
Other genes	Envelope membrane protein	*cem*A				
	Subunit of acetyl-CoA carboxylase	*acc*D				
	C-type cytochrome synthesis gene	*ccs*A				
Genes of unknown function	Conserved open reading frames	*ycf*2^**c**^	*ycf*3	*ycf*4	*ycf*15^(x2)^	

The number of encoded functional genes from the species we sequenced ranged from 135 to 138 (Table 1), which are not significantly different from the 137 reported in *A. sinensis*^26^, 142 in *A. yunnanensis* (MG656407) and 125 in *A. crassna* (MK 779,998). There are 38 tRNA genes, eight rRNA genes, and 89–92 protein coding genes in each of the cp genome (Tables 1 and 2). The IR region contains five rRNA genes and 22 other single repeat genes (*trn*A-UGC, *trn*I-CAU*, trn*I-GAU*, trn*L-CAA*, trn*L-UAG, *trn*N-GUU, *trn*R-ACG*, trn*V-GAC, *rps*7, *rps*12, *rps*15, *rpl*2, *rpl*23, *ndh*A, *ndh*B, *ndh*D, *ndh*E, *ndh*G, *ndh*H, *ndh*I, *psa*C, and *ycf*15). There are slight differences between the *Aquilaria* cp genomes such as the number of genes present, even though the cp genomes of land plants are generally considered as highly conserved^51^. Other examples like several genes appeared to be species-specific: *rps*18 in *A. malaccensis* and *A. microcarpa*, *rpl*16 in *A. hirta* and *A. malaccensis*, and *ycf*2 in *A. malaccensis* (Table 2). Presence or absence of a specific gene from several *Aquilaria* species and not others could be due to the gene being transferred to the nucleus. Gene transfer events have been observed in gene knockout experiments, such as the *rps*18 in tobacco^52^. This event most likely happened when plants are exposed to biotic and abiotic stresses, which lead to the inducing accumulation of reactive oxygen species (ROS), which activates signalling pathway when at low levels, but can cause irreparable injury to cells when produced excessively^53,54^. ROS is a normal product of plant cellular metabolism that can be affected by various types of stress^55^. ROS generated in chloroplasts can also act as signals that travel from the cp to the nucleus under stress conditions^56^. Since signals are moving from the chloroplast to the nucleus under stress condition, these transfers may also promote the transfer of chloroplast genome fragments to the nucleus where they could be incorporated into the nuclear genome^54^. Consequently, it may assist the transfer of cp gene to the nucleus^54^.

**Figure 2:**
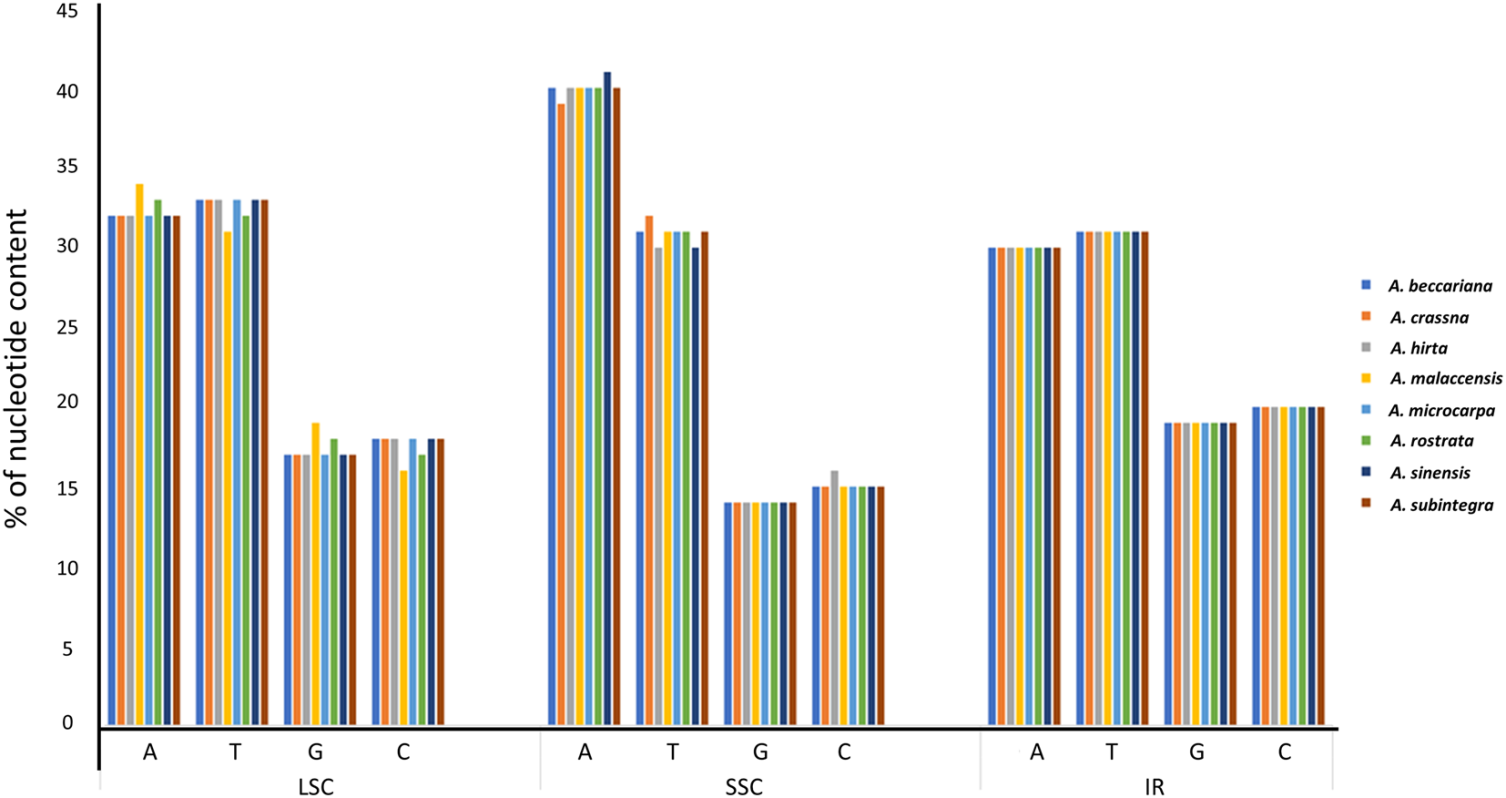
Base composition in the eight *Aquilaria* chloroplast genomes. The percentage (%) of each nucleotide (A, T, G, C) content in the three different regions, large single-copy (LSC), small single-copy (SSC) and inverted repeats (IR) region, are shown. Data for *A. malaccensis* were from Lee et al.^24^.

When comparing the eight cp genomes for base/nucleotide composition, in LSC, *A. malaccensis* has the highest percentage of A (34%) and G (18%) nucleotides, but the lowest percentage in T (32%) and C (16%) nucleotides (Fig. 2). In SSC, *A. sinensis* has the highest percentage of A (41%) nucleotide, while *A. crassna* and *A. hirta* have the highest percentage of T (32%) and C (16%) nucleotides, respectively. The overall A + T content is more than 50% when compared to G + C content (Fig. 2). This study shows that *Aquilaria* cp genomes have high levels of A + T content, a feature generally observed in many cp genomes sequences of angiosperm species^57^.

### Interspecies chloroplast genome sequence analysis

Multiple cp genome sequence alignment of the eight *Aquilaria* cp genomes with a total of 174,832 nucleotide sites revealed 697 variable (polymorphic) sites including 405 singleton variable sites (SVS) and 292 parsimony informative sites (PIS) (Table 3). There are two different categories under SVS, 403 sites with two variants (SV2V) and two sites with three variants (SV3V). Similarly, the PIS also has two variants (PIS2V) (288 sites) and three variants (PIS3V) (4 sites) (Table 3). In the SV2V category, *A. rostrata* has the highest number of SVS (nucleotide: A = 17, T = 22, G = 47, and C = 45), followed by *A. hirta* (nucleotide: A (30), T (19), G (35) and C (35) and *A. sinensis* (nucleotide: A (23), T (24), G (40) and C (28), while *A. crassna* has none (Table 4). In the PIS2V category, *A. hirta* has the highest number of PIS for nucleotide A (81), while *A. crassna* for nucleotide T (90). In summary, most of the variable sites were identified in *A. hirta*, *A. rostrata* and *A. sinensis* (Table 4). The information on SVS and PIS are useful for species identification studies and for determining phylogenetic relationships^58,59^.

**Table 3:** Variable site analysis shows the presence of singleton variable sites (SV) and parsimony informative sites (PIS) in the eight *Aquilaria* chloroplast genomes.

Number of sites	Invariable (monomorphic) sites	Variable (polymorphic) sites	Singleton variable sites (SV)	Parsimony informative sites (PIS)	SV2V	PIS2V	SV3V	PIS3V
174,832	173,720	697	405	292	403	288	2	4

*SV2V* singleton variable sites with two variants, *SV3V* singleton variable sites with three variants, *PIS2V* parsimony informative sites with two variants, and *PIS3V* parsimony informative sites with three variants.

**Table 4:** Number of nucleotides in variable site analysis among eight *Aquilaria* species.

	*Aquilaria beccariana*	*Aquilaria crassna*	*Aquilaria hirta*	*Aquilaria malaccensis*	*Aquilaria microcarpa*	*Aquilaria rostrata*	*Aquilaria sinensis*	*Aquilaria subintegra*
SV2V								
A	0	0	30	1	4	17	23	1
T	1	0	19	0	5	22	24	0
G	0	0	35	2	12	47	40	0
C	1	0	35	0	8	45	28	2
SV3V								
A	0	0	0	0	1	0	0	0
T	0	0	0	0	1	0	1	0
G	0	0	0	0	0	0	1	0
C	0	0	0	0	0	0	0	0
PIS2V								
A	76	66	81	77	71	66	79	70
T	61	90	76	60	70	86	75	85
G	69	64	68	70	67	64	65	66
C	82	68	63	81	80	72	69	67
PIS3V								
A	2	1	1	2	3	2	2	1
T	2	1	0	1	1	1	0	1
G	0	1	1	0	0	1	2	1
C	0	1	2	1	0	0	0	1

*SV2V* singleton variable sites with two variants, *SV3V* singleton variable sites with three variants, *PIS2V* parsimony informative sites with two variants, and *PIS3V* parsimony informative sites with three variants.

### IR contraction and expansion

**Figure 3:**
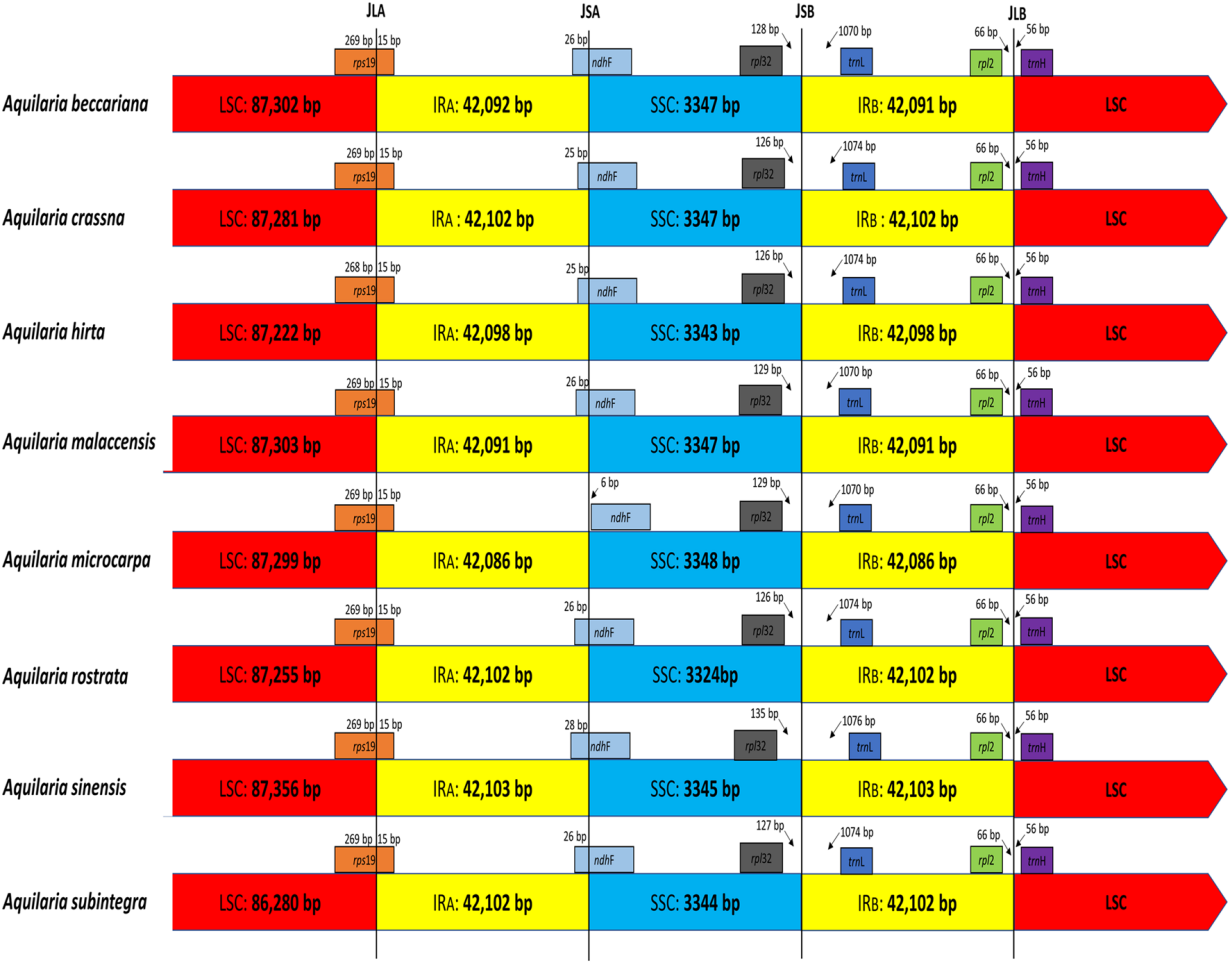
Comparison of the border regions of LSC, SSC, and IR among the eight *Aquilaria* chloroplast genomes.

Close examination of the IR/SC boundary regions among the eight *Aquilaria* species revealed three main differences (Fig. 3). Firstly, the *rps*19 gene (284 bp) is extended beyond the LSC into the IR_A_ region by 15 bp in all species. Secondly, the *ndh*f gene spans the IR_A_/SSC border, between 25 to 28 bp in the IR_A_ region and 2,211 bp in the SSC region, except in *A. microcarpa*, where it is completely in the SSC region, distanced by 6 bp from the IR_A_ region. Thirdly, in all the eight *Aquilaria* species, the *rpl*32 and *trn*L genes are in the SSC region and IR_B_ region, respectively, however with slight differences in the distance to or from the ISSC/R_B_ border. No differences were observed in the IR_B_/LSC border region; the *rpl*2 gene is in the IR_B_ region and the *trn*H is in the LSC region. In general, the IR region is one of the main reasons for a change in the size of the cp genome due to expansion, shrinkage and loss of the IR^60^.

### Large sequence repeat analyses

**Figure 4:**
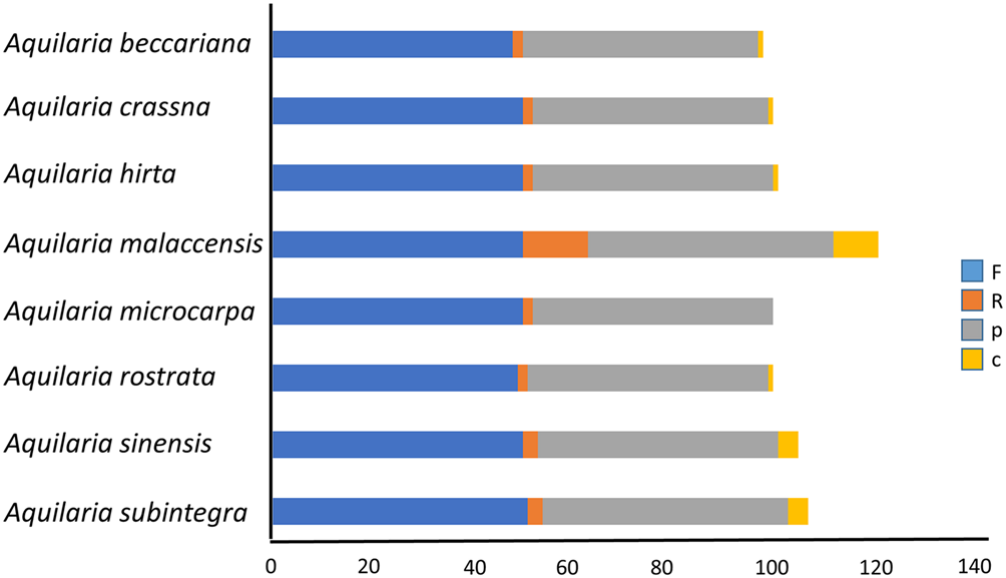
Large sequence repeat (LSR) analysis. The eight *Aquilaria* chloroplast genomes have four repeat types, which are forward (F), reverse (R), palindrome (P) and complement (C).

The large sequence repeat (LSR) of the eight *Aquilaria* cp genomes were analyzed using REPuter software. A total of 832 repeats (at least 30 bp per repeat unit with Hamming distance = 3), including forward (F), reverse (R), palindromic (P) and complement (C) repeats were identified (Table 5, and Supplementary Tables 1 to 31). In general, F repeats are the most common type detected in the *Aquilaria* cp genomes, while C repeats are the least. Among the eight species, C repeats are absent from *A. rostrata*, although it has 50 F and 2 R repeats, and 48 P repeats (Fig. 4). Large repeat sequences are informative for phylogenetic studies of *Aquilaria* species as they play important roles in cp genome evolution and may aid in future development of molecular markers^61^.

**Table 5:** Repeat sequence analysis in eight *Aquilaria* cp genomes with different hamming distance.

Species	Hamming distance = 3					Hamming distance = 2					Hamming distance = 1				
	F	R	C	P	Total	F	R	C	P	Total	F	R	C	P	Total
*A. malaccensis*	51	3	4	49	107	42	3	2	39	86	30	1	0	24	55
*A. beccariana*	50	3	4	48	105	42	3	2	38	85	30	1	0	24	55
*A. crassna*	50	2	1	48	101	41	2	0	33	76	29	1	0	24	54
*A. subintegra*	48	2	1	47	98	41	2	0	32	75	29	1	0	24	54
*A. microcarpa*	49	2	1	48	100	41	2	0	35	78	30	1	0	23	54
*A. hirta*	50	13	9	49	121	46	13	8	46	113	31	11	6	35	52
*A. sinensis*	50	2	1	47	100	43	2	0	33	78	30	1	0	24	55
*A. rostrata*	50	2	0	48	100	44	2	0	35	81	33	1	0	25	59
Total repeat sequences	398	29	21	384	832	340	29	12	291	672	242	18	6	203	379

*F* forward, *R* reverse, *C* complement, *P* palindromic matching.

Frequent variation in repeat regions in most angiosperm plants occurs due to slipped-strand mispairing and illegitimate recombination. Frequent variation in repeat regions also plays an important role in variation and sequence rearrangement in cp genomes^20,62^. In addition, the quantity of the identified repeats is sensitive to the Hamming distance used. For example, when we cut the Hamming distance from 3 to 1 (in other words rigidity was augmented), the number of repeat sequences was lowered (Table 5).

### Identification of highly variable regions within the *Aquilaria* cp genomes

**Figure 5:**
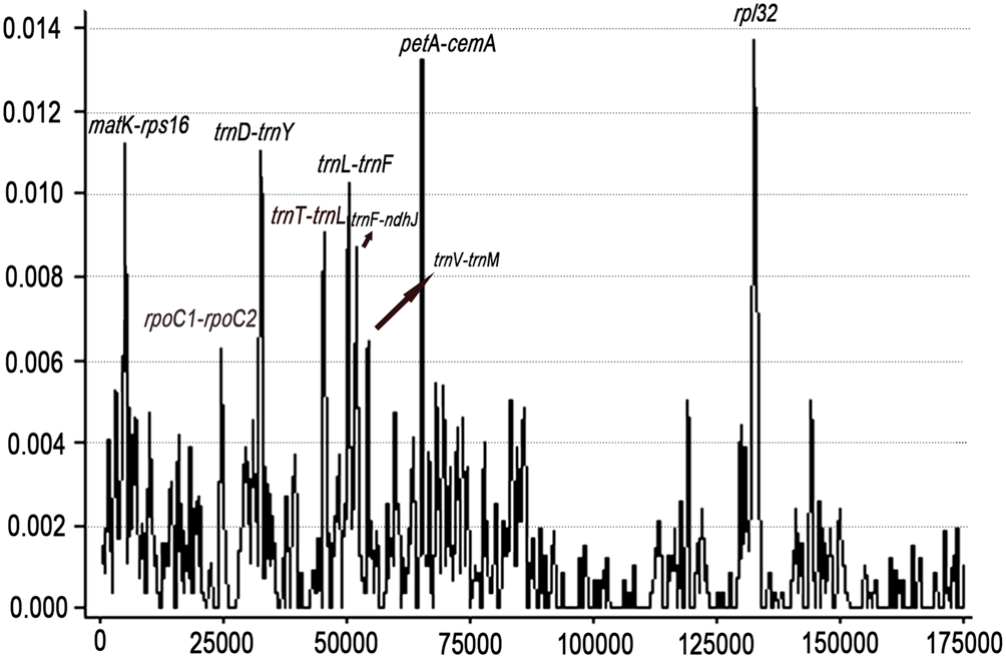
Nucleotide variability values compared between the eight chloroplast genomes of *Aquilaria* using the window sliding analysis (window length: 600 bp and step size: 200 bp). X-axis indicates the position of the midpoint of the window, while Y-axis indicates the nucleotide diversity of each window.

Using the alignment created by MAFFT and DnaSP software, the nucleotide variability (Pi) values within 600 bp window were calculated in all eight cp genomes. They are in the range from 0 to 0.01370 (Fig. 5). There are nine highly divergent regions (Pi > 0.005), divided between the intergenic spacer (IGS) region (*trn*D-*trn*Y, *trn*T-*trn*L, *trn*L-*trn*F, *trn*F-*ndh*J, *trn*V-*trn*M) and the coding sequence (CDS) regions (*mat*K-*rps*16, *rpo*C1-*rpo*C2, *pet*A-*cem*A and *rpl*32) (Fig. 5). In total, there are 144 variable sites, 72 parsimony informative sites and Pi values from 0.00630 to 0.01370, in the nine regions (Table 6). Among these, *rpl*32 has the most nucleotide variation (0.01370). Meanwhile, we found that the IR region is extremely conserved (Pi < 0.005) because highly variable region/divergent sequences were not found.

**Table 6:** Nine regions of highly variable sequences in *Aquilaria.*

No	High variable marker	Length (bp)	Variable sites	Parsimony informative sites	Nucleotide diversity (Pi)
1	*mat*K-*rps*16	605	32	18	0.01120
2	*rpo*C1-*rpo*C2	605	9	4	0.00630
3	*trn*D-*trn*Y	617	10	7	0.01102
4	*trn*T-*trn*L	601	12	9	0.00898
5	*trn*L-*trn*F	601	15	10	0.01028
6	*trn*F-*ndh*J	615	9	6	0.00870
7	*trn*V-*trn*M	633	16	3	0.00648
8	*pet*A-*cem*A	604	18	7	0.01324
9	*rpl*32	610	23	8	0.01370
Total		5,491	144	72	0.08990

### Phylogenetic analysis

**Figure 6:**
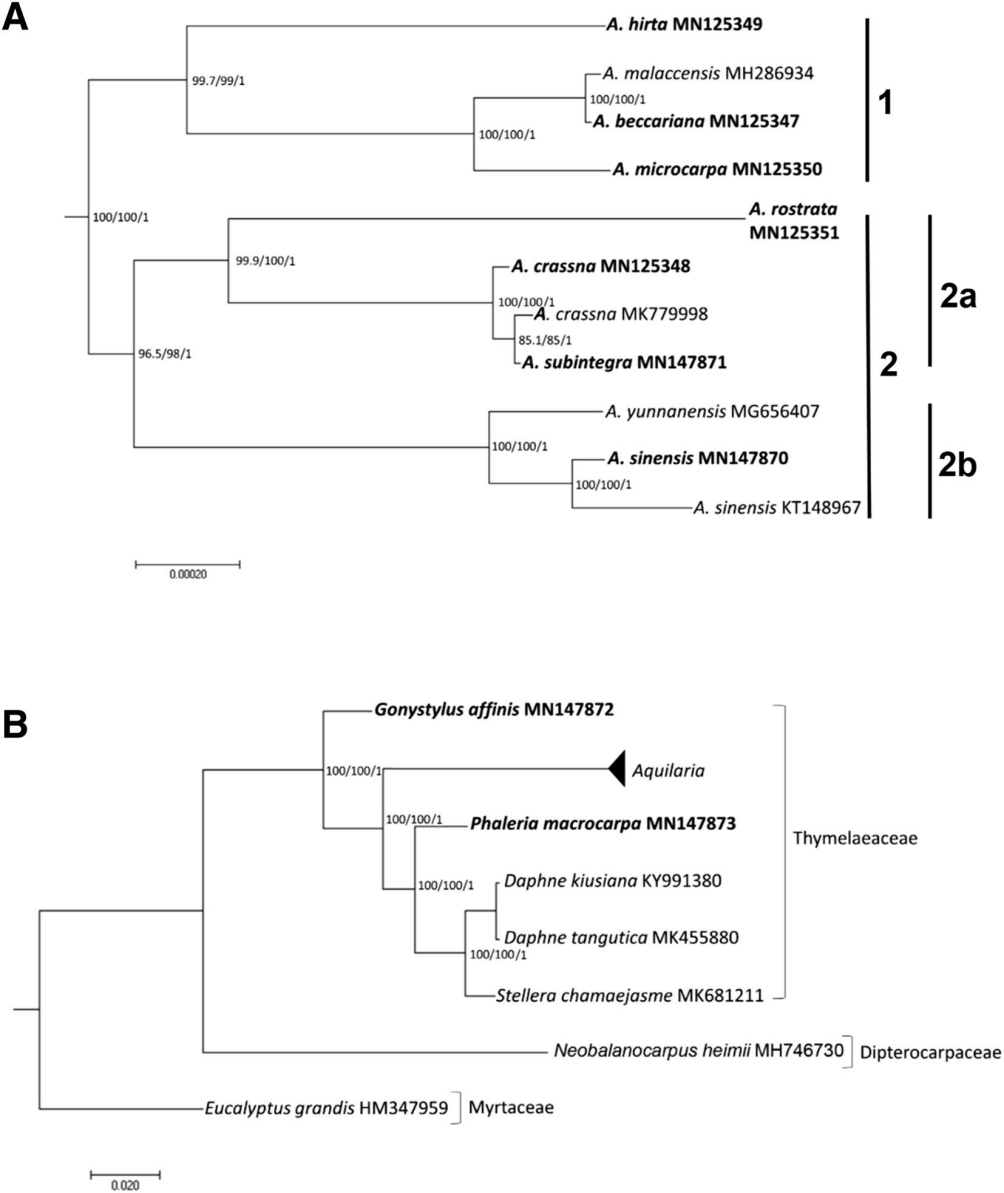
Phylogram depicting the relationships among different *Aquilaria* species (**A**), and molecular placement of *Aquilaria* genus in the family Thymelaeaceae (**B**), estimated using maximum likelihood (ML) and bayesian inference (BI) analysis in IQ-TREE and MrBayes. The data set was partitioned by the optimal scheme identified using the ModelFinder option of IQ-TREE. The bootstrap was set at 2000 replicates and the support value is indicated at each branch, where the first number indicates SH-aLRT value, the second number indicates UFboot value and the third number indicates posterior probability (pp) value. Five closely related taxa, *Daphne kiusiana* (KY991380), *Daphne tangutica* (MK455880), *Gonystylus affinis* (MN147872), *Phaleria macrocarpa* (MN147873), and *Stellera chamaejasme* (MK681211), and two outgroups, *Eucalyptus grandis* (HM347959) and *Neobalanocarpus heimii* (MH746730), were included. Species sequenced in this study are in bold. GenBank accession numbers are indicated for each species.

For construction of phylogenetic trees, the Maximum Likelihood (ML) analyses were performed via IQ-TREE v.1.4.2 software^41^ and Bayesian Inference (BI) analyses were performed via MrBayes v3.2.7 software^46^ using the complete cp genomes of 11 accessions (nine *Aquilaria* species), and the results are summarized in Fig. 6. Similar phylogenetic topologies structures were found in the ML and BI nucleic acid analyses. The nine *Aquilaria* species are diverged into two major clades (Clade 1 and 2) showing a paraphyletic relationship, with a strong support as indicated from the high bootstrap values for SH-aLRT and UFBoot and posterior probability values (100%, 100%, and 1, respectively) (Fig. 6A). Clade 1 has three species of Malay Peninsula origin (*A. hirta*, *A. beccariana* and *A. malaccensis*) and one species of Borneo origin (*A. microcarpa*). They come from a recent common ancestor (99.7%, 99%, and 1). The branch is considered reliable when the support value of SH-aLRT, UFBoot, and posterior probability values are > 80%, > 95% and > 0.75, respectively^46,63^. Clade 2 is further diverged into two sub-clades. Sub-clade 2a shows that *A. rostrata* is genetically distanced from *A. crassna* and *A. subintegra* (99.9%, 100%, and 1). *Aquilaria rostrata* is an endemic species of Malay Peninsula, confined to high altitudes^64^. We also showed that *A. crassna* (MK779998), which originated from Cambodia and *A. subintegra* (MN147871) from Thailand are sisters. However, the low support values (85.1%, 85%, and 1) suggest that local hybridization might have occurred^65^, although this needs further investigation. We have observed that in the field, *A. crassna* and *A. subintegra*, have very similar morphological features^5^. Interestingly, when comparing the polymorphic sites, except for the SV2V category, these two species have the same number of nucleotides in the PIS3V category (Table 4). Meanwhile, sub-clade 2b shows that two *Aquilaria* species of China origin (*A. sinensis* and *A. yunnanensis*) are grouped together with strong support values (100%, 100%, and 1) (Fig. 6A). We conclude that the phylogenetic positions within the *Aquilaria* species reported here corresponds well with their natural geographic distribution pattern. When compared to the recent *Aquilaria* phylogenetic tree constructed using a concatenated dataset of five cp gene sequence (*mat*K, *rbc*L, *trn*L intron, *trn*L-*trn*F, and *psb*C-*trn*S)^11^, a consistent clustering pattern was observed. However, our cp genome-ML tree has a higher statistical support. This shows that comparative analysis using complete cp genome reveals greater abundance in informative characters when compared to the short gene fragments in *Aquilaria*.

Figure 6B exhibits *Aquilaria*’s position in relation to other member taxa in the family Thymelaeaceae. All *Aquilaria* species clustered into a strongly supported clade (100% ,100%, and 1) after *G. affinis*. *Aquilaria* is the first to diverge from *Gonystylu*s followed by *Phaleria* and *Daphne* (Fig. 6B). Both latter taxa are under the Daphneae tribe, placed as sister to the Aquilarieae tribe in the subfamily Thymelaeoideae. Our findings are in agreement with the classification system by Herber (2003)^66^, who proposed two major subfamilies for Thymelaeaceae, Octolepidoideae (*Gonystylus*) and Thymelaeoideae (s.l.). The latter subfamily is further divided into tribes Aquilarieae (*Aquilaria*), Daphneae (*Phaleria*, *Daphne*, and *Stellera*) and Synandrodaphneae. Similarly, our results compliment the classification system of Domke (1934)^67^, as shown by Beaumont et al.^68^ through a phylogenetic analysis involving 143 specimens from members of the Thymelaeaceae family and the combined dataset of *rbc*L + *trn*L-*trn*F + ITS. *Aquilaria*, placed under the subfamily Aquilarioideae, is shown to evolve after the Gonystyloideae (*Gonystylus*), and sister to Thymelaeoideae (*Phaleria*, *Daphne*, and *Stellera*)^66^.

## Conclusion

In this study, we report new complete cp genomes sequences from seven *Aquilaria* species and analyzed these genomes including another, which we recently published. The eight *Aquilaria* cp genomes were similar in genome content, structure, and gene order. Comparison of the eight *Aquilaria* cp genomes revealed 832 LSR and nine divergent regions (*trn*D-*trn*Y, *trn*T-*trn*L, *trn*L-*trn*F, *trn*F-*ndh*J, *trn*V-*trn*M*, mat*K-*rps*16, *rpo*C1-*rpo*C2, *pet*A-*cem*A and *rpl*32). Both ML and BI phylogenetic analyses strongly supported the phylogenetic positions within the *Aquilaria* species and their natural geographic distribution pattern. We have successfully revealed the complete cp genome sequences for eight *Aquilaria* species, in which five were native to Malaysia. Future studies should identify potential molecular markers to provide a clear discrimination between these important and closely related genetic resources.

## Supplementary information

Supplementary Information.
