# Author Correction: Comparison of eight complete chloroplast genomes of the endangered *Aquilaria* tree species (Thymelaeaceae) and their phylogenetic relationships

**DOI:** 10.1038/s41598-021-88638-1

**Published:** 2021-04-22

**Authors:** Muhammad Syahmi Hishamuddin, Shiou Yih Lee, Wei Lun Ng, Shairul Izan Ramlee, Dhilia Udie Lamasudin, Rozi Mohamed

**Affiliations:** 1 Forest Biotechnology Laboratory, Department of Forestry Science and Biodiversity, Faculty of Forestry and Environment, Universiti Putra Malaysia, 43400 Serdang, Selangor Malaysia; 2 China‑ASEAN College of Marine Sciences, Xiamen University Malaysia, 43900 Sepang, Selangor Malaysia; 3 Department of Crop Science, Faculty of Agriculture, Universiti Putra Malaysia, 43400 Serdang, Selangor Malaysia; 4 Department of Cell and Molecular Biology, Faculty of Biotechnology and Biomolecular Sciences, Universiti Putra Malaysia, 43400 Serdang, Selangor Malaysia; 5 Halal Products Research Institute, Universiti Putra Malaysia, 43400 Serdang, Selangor Malaysia

Correction to: *Scientific Reports* 10.1038/s41598-020-70030-0, published online 03 August 2020

This Article contains errors in the gene names.

This has resulted in errors in Figure 5, and in Table 6. The correct Figure 5 appears below as Figure 1, and the correct Table 6 appears below as Table 1.

**Figure 1:**
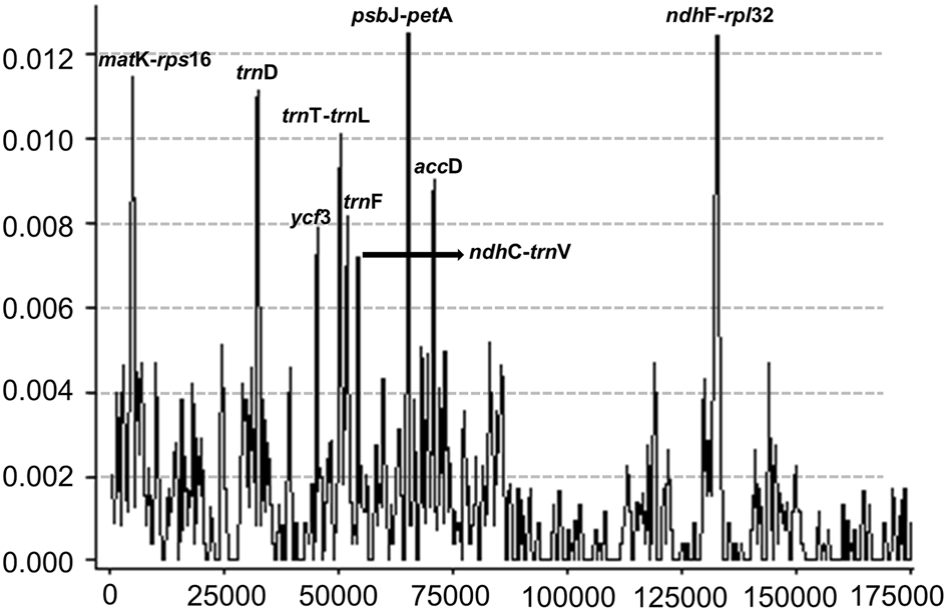
A correct version of the original Figure 5.

**Table 1:** A correct version of the original Table 6.

No	High variable marker	Length (bp)	Variable sites	Parsimony informative sites	Nucleotide diversity (Pi)
1	*mat*K-*rps*16	605	17	10	0.01093
2	*trn*D	1,017	27	11	0.01077
3	*ycf*3	1,022	15	2	0.00792
4	*trn*T-*trn*L	801	28	9	0.01012
5	*trn*F	619	14	6	0.00815
6	*ndh*C-*trn*V	1,033	16	1	0.00706
7	*psb*J-*pet*A	804	34	14	0.01208
8	*acc*D	865	20	10	0.00905
9	*ndh*F-*rpl*32	1,328	36	12	0.01244
**Total**		**8,094**	**207**	**75**	**0.08852**

Additionally, in the Results and discussion section under subheading ‘Identification of highly variable regions within the *Aquilaria* cp genomes’,

“They are in the range from 0 to 0.01370 (Fig. 5). There are nine highly divergent regions (Pi > 0.005), divided between the intergenic spacer (IGS) region (*trn*D-*trn*Y, *trn*T-*trn*L, *trn*L-*trn*F, *trn*F*ndh*J, *trn*V-*trn*M) and the coding sequence (CDS) regions (*mat*K-*rps*16, *rpo*C1-*rpo*C2, *pet*A-*cem*A and *rpl*32) (Fig. 5). In total, there are 144 variable sites, 72 parsimony informative sites and Pi values from 0.00630 to 0.01370, in the nine regions (Table 6). Among these, *rpl*32 has the most nucleotide variation (0.01370). Meanwhile, we found that the IR region is extremely conserved (Pi < 0.005) because highly variable region/divergent sequences were not found.”

should read:

“They are in the range from 0 to 0.01244 (Fig. 5). There are nine highly divergent regions (Pi > 0.006), divided between the intergenic spacer (IGS) region (*mat*K-*rps*16, *ndh*C-*trn*V, *ndh*F-*rpl*32, *psb*J-*pet*A and *trn*T-*trn*L) and the coding sequence (CDS) regions (*acc*D, *trn*D, *trn*F and *ycf*3) (Fig. 5). In total, there are 207 variable sites, 75 parsimony informative sites and Pi values from 0.00706 to 0.01244, in the nine regions (Table 6). Among these, *ndh*F-*rpl*32 has the most nucleotide variation (0.01244). Meanwhile, we found that the IR region is extremely conserved (Pi < 0.006) because highly variable region/divergent sequences were not found.”

Finally, in the Conclusion section,

“Comparison of the eight *Aquilaria* cp genomes revealed 832 LSR and nine divergent regions (*trn*D-*trn*Y, *trn*T-*trn*L, *trn*L-*trn*F, *trn*F-*ndh*J, *trn*V-*trn*M*, mat*K-*rps*16, *rpo*C1-*po*C2, *pet*A-*cem*A and *rpl*32).”

should read:

“Comparison of the eight *Aquilaria* cp genomes revealed 832 LSR and nine divergent regions (*mat*K-*rps*16, *trn*D, *ycf*3, *trn*T-*trn*L, *trn*F, *ndh*C-*trn*V, *psb*J-*pet*A, *acc*D, and *ndh*F-*rpl*32).”

