# Supplementary material for: Comparison of eight complete chloroplast genomes of the endangered Aquilaria tree species (Thymelaeaceae) and their phylogenetic relationships
Source: Sci Rep. 2020 Aug 3;10:13034. doi: 10.1038/s41598-020-70030-0 (PMC7400740; doi:10.1038/s41598-020-70030-0)
Supplement: Supplementary file 1 — Supplementary Information. [file 41598_2020_70030_MOESM1_ESM.pdf]

Comparison of eight complete chloroplast genomes of endangered *Aquilaria* tree species (Thymelaeaceae) and phylogenetic relationship analysis

Muhammad Syahmi Hishamuddin[1], Shiou Yih Lee[1], Wei Lun Ng[2], Shairul Izan Ramlee[3], Dhilia Udie Lamasudin[4,5], Rozi Mohamed[1,*]

[1]Forest Biotechnology Laboratory, Department of Forest Management, Faculty of Forestry, Universiti Putra Malaysia, 43400 UPM Serdang, Selangor, Malaysia.

[2]China-ASEAN College of Marine Sciences, Xiamen University Malaysia, Sepang, Selangor, Malaysia

[3]Department of Crop Science, Faculty of Agriculture, Universiti Putra Malaysia, 43400 UPM Serdang, Selangor, Malaysia

[4]Department of Cell and Molecular Biology, Faculty of Biotechnology and Biomolecular Sciences, Universiti Putra Malaysia, 43400 UPM Serdang, Selangor, Malaysia

[5]Halal Products Research Institute, Universiti Putra Malaysia, 43400 UPM Serdang, Selangor, Malaysia

* E-mail: rozimohd@upm.edu.my

Supplementary Table 1: Forward repeat in *Aquilaria beccariana* chloroplast genome

No	Repeat length of the first part	Starting position of the first part	Match direction	Repeat length of the second part	Starting position of the second part	Distance of this repeat	Calculated e-value of this repeat
1.	163	1629	F	163	71966	-3	1.20E-81
2.	156	1642	F	156	71979	-3	1.73E-77
3.	78	128147	F	78	128227	-3	1.93E-31
4.	78	133827	F	78	133907	-3	1.93E-31
5.	70	113388	F	70	113457	-1	1.30E-30
6.	70	148605	F	70	148674	-1	1.30E-30
7.	62	148613	F	62	148682	0	4.04E-28
8.	64	133841	F	64	133921	-1	4.85E-27
9.	62	70232	F	62	70292	-1	7.52E-26
10.	63	128167	F	63	128247	-3	1.08E-22
11.	59	112671	F	59	112692	-2	3.98E-22
12.	59	149381	F	59	149402	-2	3.98E-22
13.	57	70209	F	57	70227	-2	5.95E-21
14.	51	149389	F	51	149410	-1	2.59E-19
15.	50	70213	F	50	70291	-1	1.02E-18
16.	44	113522	F	44	113564	0	2.78E-17
17.	44	148524	F	44	148566	0	2.78E-17
18.	41	95909	F	41	95927	0	1.78E-15
19.	41	166164	F	41	166182	0	1.78E-15
20.	43	97901	F	43	164188	-1	1.43E-14
21.	38	112671	F	38	112713	-1	1.30E-11
22.	38	113532	F	38	113595	-1	1.30E-11
23.	38	148499	F	38	148562	-1	1.30E-11
24.	38	149381	F	38	149423	-1	1.30E-11
25.	41	41195	F	41	43419	-2	1.31E-11
26.	41	101940	F	41	140171	-2	1.31E-11
27.	41	121920	F	41	160151	-2	1.31E-11
28.	39	70209	F	39	70245	-2	1.90E-10
29.	36	46454	F	36	101943	-1	1.97E-10
30.	41	1526	F	41	71859	-3	5.12E-10
31.	41	1535	F	41	71868	-3	5.12E-10
32.	41	113939	F	41	113978	-3	5.12E-10
33.	41	148113	F	41	148152	-3	5.12E-10
34.	38	94602	F	38	94620	-2	7.20E-10
35.	38	167474	F	38	167492	-2	7.20E-10
36.	31	59854	F	31	59884	0	1.86E-09
37.	34	70250	F	34	70292	-1	2.97E-09
38.	34	113574	F	34	113595	-1	2.97E-09
39.	34	148503	F	34	148524	-1	2.97E-09
40.	39	113546	F	39	113609	-3	7.02E-09
41.	39	148484	F	39	148547	-3	7.02E-09
42.	30	149410	F	30	149431	0	7.46E-09
43.	36	70209	F	36	70305	-2	1.03E-08
44.	31	70292	F	31	70310	-1	1.73E-07
45.	31	112681	F	31	112723	-1	1.73E-07
46.	36	46454	F	36	140174	-3	3.51E-07

47.	36	113532	F	36	113553	-3	3.51E-07
48.	36	148543	F	36	148564	-3	3.51E-07
49.	30	46463	F	30	101952	-1	6.71E-07
50.	34	113553	F	34	113574	-3	4.71E-06

Supplementary Table 2: Reverse repeat in *Aquilaria beccariana* chloroplast genome

No	Repeat length of the first part	Starting position of the first part	Match direction	Repeat length of the second part	Starting position of the second part	Distance of this repeat	Calculated e-value of this repeat
1.	31	33569	R	31	33569	0	1.86E-09
2.	35	33568	R	35	33568	-2	3.90E-08
3.	31	33571	R	31	33571	-2	7.80E-06

Supplementary Table 3: Palindrome repeat in *Aquilaria beccariana* chloroplast genome

No	Repeat length of the first part	Starting position of the first part	Match direction	Repeat length of the second part	Starting position of the second part	Distance of this repeat	Calculated e-value of this repeat
1.	78	128227	P	78	133907	-3	1.93E-31
2.	70	113388	P	70	148605	-1	1.30E-30
3.	70	113457	P	70	148674	-1	1.30E-30
4.	54	10415	P	54	10415	0	2.65E-23
5.	63	128167	P	63	133822	-3	1.08E-22
6.	63	128247	P	63	133902	-3	1.08E-22
7.	59	112671	P	59	149381	-2	3.98E-22
8.	59	112692	P	59	149402	-2	3.98E-22
9.	44	113522	P	44	148524	0	2.78E-17
10.	44	113564	P	44	148566	0	2.78E-17
11.	41	95909	P	41	166164	0	1.78E-15
12.	41	95927	P	41	166182	0	1.78E-15
13.	49	77049	P	49	77049	-3	1.35E-14
14.	43	97901	P	43	97901	-1	1.43E-14
15.	43	164188	P	43	164188	-1	1.43E-14
16.	38	112671	P	38	149381	-1	1.30E-11
17.	38	112713	P	38	149423	-1	1.30E-11
18.	38	113532	P	38	148499	-1	1.30E-11
19.	38	113595	P	38	148562	-1	1.30E-11
20.	41	101940	P	41	121920	-2	1.31E-11
21.	41	140171	P	41	160151	-2	1.31E-11
22.	36	46454	P	36	160153	-1	1.97E-10
23.	41	38603	P	41	38603	-3	5.12E-10
24.	41	113939	P	41	148113	-3	5.12E-10
25.	41	113978	P	41	148152	-3	5.12E-10
26.	38	94602	P	38	167474	-2	7.20E-10
27.	38	94620	P	38	167492	-2	7.20E-10
28.	34	113574	P	34	148503	-1	2.97E-09
29.	34	113595	P	34	148524	-1	2.97E-09
30.	39	113546	P	39	148484	-3	7.02E-09
31.	39	113609	P	39	148547	-3	7.02E-09
32.	31	112681	P	31	149378	-1	1.73E-07
33.	31	112723	P	31	149420	-1	1.73E-07
34.	36	46454	P	36	121922	-3	3.51E-07
35.	36	113532	P	36	148543	-3	3.51E-07
36.	36	113553	P	36	148564	-3	3.51E-07
37.	30	8660	P	30	47899	-1	6.71E-07
38.	30	46463	P	30	160150	-1	6.71E-07
39.	32	33569	P	32	33569	-2	2.08E-06
40.	32	33570	P	32	33570	-2	2.08E-06
41.	34	113553	P	34	148524	-3	4.71E-06
42.	34	113574	P	34	148545	-3	4.71E-06
43.	31	69259	P	31	69297	-2	7.80E-06
44.	31	112702	P	31	149378	-2	7.80E-06

45.	31	112723	P	31	149399	-2	7.80E-06
46.	30	33569	P	30	33569	-2	2.92E-05
47.	30	33572	P	30	33572	-2	2.92E-05
48.	32	4879	P	32	4917	-3	6.24E-05

Supplementary Table 4: Complement repeat in *Aquilaria beccariana* chloroplast genome

No	Repeat length of the first part	Starting position of the first part	Match direction	Repeat length of the second part	Starting position of the second part	Distance of this repeat	Calculated e-value of this repeat
1.	32	33569	C	32	33570	-2	2.08E-06
2.	30	33569	C	30	33572	-2	2.92E-05
3.	30	30482	C	30	30484	-3	8.17E-04
4.	30	33569	C	30	33574	-3	8.17E-04

Supplementary Table 5: Forward repeat in *Aquilaria crassna* chloroplast genome

No	Repeat length of the first part	Starting position of the first part	Match direction	Repeat length of the second part	Starting position of the second part	Distance of this repeat	Calculated e-value of this repeat
1.	152	1629	F	152	71945	-2	2.72E-77
2.	150	1642	F	150	71958	-3	6.28E-74
3.	137	1661	F	137	71977	-3	3.21E-66
4.	78	128134	F	78	128214	-3	1.93E-31
5.	78	133819	F	78	133899	-3	1.93E-31
6.	70	113375	F	70	113444	-1	1.30E-30
7.	70	148597	F	70	148666	-1	1.30E-30
8.	62	148605	F	62	148674	0	4.04E-28
9.	64	133833	F	64	133913	-1	4.85E-27
10.	63	70209	F	63	70269	-1	1.91E-26
11.	62	112658	F	62	112679	-2	6.88E-24
12.	62	149370	F	62	149391	-2	6.88E-24
13.	63	128154	F	63	128234	-3	1.08E-22
14.	51	149381	F	51	149402	-1	2.59E-19
15.	44	113509	F	44	113551	0	2.78E-17
16.	44	148516	F	44	148558	0	2.78E-17
17.	41	95889	F	41	95907	0	1.78E-15
18.	41	166163	F	41	166181	0	1.78E-15
19.	41	112658	F	41	112700	-1	2.19E-13
20.	41	149370	F	41	149412	-1	2.19E-13
21.	38	113519	F	38	113582	-1	1.30E-11
22.	38	148491	F	38	148554	-1	1.30E-11
23.	41	41181	F	41	43405	-2	1.31E-11
24.	41	101927	F	41	140163	-2	1.31E-11
25.	41	121907	F	41	160143	-2	1.31E-11
26.	39	70205	F	39	70223	-2	1.90E-10
27.	36	46446	F	36	101930	-1	1.97E-10
28.	41	1526	F	41	71838	-3	5.12E-10
29.	41	1535	F	41	71847	-3	5.12E-10
30.	41	113926	F	41	113965	-3	5.12E-10
31.	41	148105	F	41	148144	-3	5.12E-10
32.	38	94582	F	38	94600	-2	7.20E-10
33.	38	167473	F	38	167491	-2	7.20E-10
34.	31	59849	F	31	59879	0	1.86E-09
35.	31	112668	F	31	112710	0	1.86E-09
36.	34	70228	F	34	70270	-1	2.97E-09
37.	34	113561	F	34	113582	-1	2.97E-09
38.	34	148495	F	34	148516	-1	2.97E-09
39.	39	113533	F	39	113596	-3	7.02E-09
40.	39	148476	F	39	148539	-3	7.02E-09
41.	30	149402	F	30	149423	0	7.46E-09
42.	36	70205	F	36	70283	-2	1.03E-08
43.	31	70270	F	31	70288	-1	1.73E-07
44.	31	112689	F	31	112710	-1	1.73E-07

45.	36	46446	F	36	140166	-3	3.51E-07
46.	36	113519	F	36	113540	-3	3.51E-07
47.	36	148535	F	36	148556	-3	3.51E-07
48.	30	46455	F	30	101939	-1	6.71E-07
49.	34	113540	F	34	113561	-3	4.71E-06
50.	34	148516	F	34	148537	-3	4.71E-06

Supplementary Table 6: Reverse repeat in *Aquilaria crassna* chloroplast genome

No	Repeat length of the first part	Starting position of the first part	Match direction	Repeat length of the second part	Starting position of the second part	Distance of this repeat	Calculated e-value of this repeat
1.	30	33551	R	30	33554	-1	6.71E-07
2.	31	33551	R	31	33551	-2	7.80E-06

Supplementary Table 7: Palindrome repeat in *Aquilaria crassna* chloroplast genome

No	Repeat length of the first part	Starting position of the first part	Match direction	Repeat length of the second part	Starting position of the second part	Distance of this repeat	Calculated e-value of this repeat
1.	78	128214	P	78	133899	-3	1.93E-31
2.	70	113375	P	70	148597	-1	1.30E-30
3.	70	113444	P	70	148666	-1	1.30E-30
4.	62	112658	P	62	149370	-2	6.88E-24
5.	62	112679	P	62	149391	-2	6.88E-24
6.	54	10405	P	54	10405	0	2.65E-23
7.	63	128154	P	63	133814	-3	1.08E-22
8.	63	128234	P	63	133894	-3	1.08E-22
9.	44	113509	P	44	148516	0	2.78E-17
10.	44	113551	P	44	148558	0	2.78E-17
11.	41	95889	P	41	166163	0	1.78E-15
12.	41	95907	P	41	166181	0	1.78E-15
13.	49	77028	P	49	77028	-3	1.35E-14
14.	41	112658	P	41	149370	-1	2.19E-13
15.	41	112700	P	41	149412	-1	2.19E-13
16.	38	113519	P	38	148491	-1	1.30E-11
17.	38	113582	P	38	148554	-1	1.30E-11
18.	41	101927	P	41	121907	-2	1.31E-11
19.	41	140163	P	41	160143	-2	1.31E-11
20.	36	46446	P	36	160145	-1	1.97E-10
21.	41	38585	P	41	38585	-3	5.12E-10
22.	41	113926	P	41	148105	-3	5.12E-10
23.	41	113965	P	41	148144	-3	5.12E-10
24.	38	94582	P	38	167473	-2	7.20E-10
25.	38	94600	P	38	167491	-2	7.20E-10
26.	31	112668	P	31	149370	0	1.86E-09
27.	31	112710	P	31	149412	0	1.86E-09
28.	34	113561	P	34	148495	-1	2.97E-09
29.	34	113582	P	34	148516	-1	2.97E-09
30.	39	113533	P	39	148476	-3	7.02E-09
31.	39	113596	P	39	148539	-3	7.02E-09
32.	31	112689	P	31	149370	-1	1.73E-07
33.	31	112710	P	31	149391	-1	1.73E-07
34.	36	46446	P	36	121909	-3	3.51E-07
35.	36	113519	P	36	148535	-3	3.51E-07
36.	36	113540	P	36	148556	-3	3.51E-07
37.	30	8651	P	30	47889	-1	6.71E-07
38.	30	46455	P	30	160142	-1	6.71E-07
39.	34	113540	P	34	148516	-3	4.71E-06
40.	34	113561	P	34	148537	-3	4.71E-06
41.	31	69255	P	31	69293	-2	7.80E-06
42.	32	118502	P	32	118548	-3	6.24E-05
43.	32	126692	P	32	135319	-3	6.24E-05
44.	32	126760	P	32	135387	-3	6.24E-05

45.	32	143531	P	32	143577	-3	6.24E-05
46.	31	95881	P	31	166163	-3	2.26E-04
47.	31	95917	P	31	166199	-3	2.26E-04
48.	31	96529	P	31	96529	-3	2.26E-04

Supplementary Table 8: Complement repeat in *Aquilaria crassna* chloroplast genome

No	Repeat length of the first part	Starting position of the first part	Match direction	Repeat length of the second part	Starting position of the second part	Distance of this repeat	Calculated e-value of this repeat
1.	30	30462	C	30	30464	-3	8.17E-04

Supplementary Table 9: Forward repeat in *Aquilaria hirta* chloroplast genome

No	Repeat length of the first part	Starting position of the first part	Match direction	Repeat length of the second part	Starting position of the second part	Distance of this repeat	Calculated e-value of this repeat
1.	163	1629	F	163	71892	-3	1.20E-81
2.	156	1642	F	156	71905	-3	1.73E-77
3.	78	128074	F	78	128154	-3	1.93E-31
4.	78	133750	F	78	133830	-3	1.93E-31
5.	70	113315	F	70	113384	-1	1.29E-30
6.	70	148528	F	70	148597	-1	1.29E-30
7.	62	148536	F	62	148605	0	4.04E-28
8.	64	133764	F	64	133844	-1	4.85E-27
9.	62	112598	F	62	112619	-2	6.87E-24
10.	62	149301	F	62	149322	-2	6.87E-24
11.	63	128094	F	63	128174	-3	1.08E-22
12.	51	149312	F	51	149333	-1	2.59E-19
13.	44	113449	F	44	113491	0	2.78E-17
14.	44	148447	F	44	148489	0	2.78E-17
15.	49	70168	F	49	70228	-2	2.87E-16
16.	41	95829	F	41	95847	0	1.78E-15
17.	41	166094	F	41	166112	0	1.78E-15
18.	41	112598	F	41	112640	-1	2.18E-13
19.	41	149301	F	41	149343	-1	2.18E-13
20.	37	33553	F	37	33555	0	4.55E-13
21.	35	33553	F	35	33557	0	7.28E-12
22.	38	113459	F	38	113522	-1	1.30E-11
23.	38	148422	F	38	148485	-1	1.30E-11
24.	41	41207	F	41	43431	-2	1.31E-11
25.	41	101867	F	41	140094	-2	1.31E-11
26.	41	121847	F	41	160074	-2	1.31E-11
27.	33	33553	F	33	33559	0	1.16E-10
28.	36	46466	F	36	101870	-1	1.96E-10
29.	41	1526	F	41	71785	-3	5.11E-10
30.	41	1535	F	41	71794	-3	5.11E-10
31.	41	113866	F	41	113905	-3	5.11E-10
32.	41	148036	F	41	148075	-3	5.11E-10
33.	38	94522	F	38	94540	-2	7.19E-10
34.	38	167404	F	38	167422	-2	7.19E-10
35.	35	70172	F	35	70214	-1	7.64E-10
36.	31	33553	F	31	33561	0	1.86E-09
37.	31	59857	F	31	59887	0	1.86E-09
38.	31	112608	F	31	112650	0	1.86E-09
39.	34	113501	F	34	113522	-1	2.97E-09
40.	34	148426	F	34	148447	-1	2.97E-09
41.	39	113473	F	39	113536	-3	7.01E-09
42.	39	148407	F	39	148470	-3	7.01E-09
43.	30	149333	F	30	149354	0	7.45E-09
44.	36	33546	F	36	33556	-2	1.03E-08

45.	34	33546	F	34	33558	-2	1.47E-07
46.	31	70215	F	31	70233	-1	1.73E-07
47.	31	112629	F	31	112650	-1	1.73E-07
48.	36	46466	F	36	140097	-3	3.51E-07
49.	36	113459	F	36	113480	-3	3.51E-07
50.	36	148466	F	36	148487	-3	3.51E-07

Supplementary Table 10: Reverse repeat in *Aquilaria hirta* chloroplast genome

No	Repeat length of the first part	Starting position of the first part	Match direction	Repeat length of the second part	Starting position of the second part	Distance of this repeat	Calculated e-value of this repeat
1.	41	33552	R	41	33552	0	1.78E-15
2.	37	33553	R	37	33553	0	4.55E-13
3.	37	33555	R	37	33555	0	4.55E-13
4.	35	33553	R	35	33553	0	7.28E-12
5.	35	33557	R	35	33557	0	7.28E-12
6.	33	33553	R	33	33553	0	1.16E-10
7.	33	33559	R	33	33559	0	1.16E-10
8.	31	33553	R	31	33553	0	1.86E-09
9.	31	33561	R	31	33561	0	1.86E-09
10.	34	33546	R	34	33553	-2	1.47E-07
11.	31	33561	R	31	33563	-1	1.73E-07
12.	32	33546	R	32	33553	-2	2.08E-06
13.	31	33563	R	31	33563	-2	7.80E-06

Supplementary Table 11: Palindrome repeat in *Aquilaria hirta* chloroplast genome

No	Repeat length of the first part	Starting position of the first part	Match direction	Repeat length of the second part	Starting position of the second part	Distance of this repeat	Calculated e-value of this repeat
1.	78	128074	P	78	133750	-3	1.93E-31
2.	78	128154	P	78	133830	-3	1.93E-31
3.	70	113315	P	70	148528	-1	1.29E-30
4.	70	113384	P	70	148597	-1	1.29E-30
5.	62	112598	P	62	149301	-2	6.87E-24
6.	62	112619	P	62	149322	-2	6.87E-24
7.	54	10404	P	54	10404	0	2.65E-23
8.	63	128094	P	63	133745	-3	1.08E-22
9.	63	128174	P	63	133825	-3	1.08E-22
10.	44	113449	P	44	148447	0	2.78E-17
11.	44	113491	P	44	148489	0	2.78E-17
12.	41	95829	P	41	166094	0	1.78E-15
13.	41	95847	P	41	166112	0	1.78E-15
14.	49	76975	P	49	76975	-3	1.35E-14
15.	38	33553	P	38	33553	0	1.14E-13
16.	38	33554	P	38	33554	0	1.14E-13
17.	41	112598	P	41	149301	-1	2.18E-13
18.	41	112640	P	41	149343	-1	2.18E-13
19.	44	49061	P	44	49061	-2	2.36E-13
20.	36	33553	P	36	33553	0	1.82E-12
21.	36	33556	P	36	33556	0	1.82E-12
22.	38	113459	P	38	148422	-1	1.30E-11
23.	38	113522	P	38	148485	-1	1.30E-11
24.	41	101867	P	41	121847	-2	1.31E-11
25.	41	140094	P	41	160074	-2	1.31E-11
26.	34	33553	P	34	33553	0	2.91E-11
27.	34	33558	P	34	33558	0	2.91E-11
28.	43	38600	P	43	38600	-3	3.70E-11
29.	36	46466	P	36	160076	-1	1.96E-10
30.	32	33553	P	32	33553	0	4.66E-10
31.	32	33560	P	32	33560	0	4.66E-10
32.	41	113866	P	41	148036	-3	5.11E-10
33.	41	113905	P	41	148075	-3	5.11E-10
34.	38	94522	P	38	167404	-2	7.19E-10
35.	38	94540	P	38	167422	-2	7.19E-10
36.	31	112608	P	31	149301	0	1.86E-09
37.	31	112650	P	31	149343	0	1.86E-09
38.	34	113501	P	34	148426	-1	2.97E-09
39.	34	113522	P	34	148447	-1	2.97E-09
40.	39	113473	P	39	148407	-3	7.01E-09
41.	39	113536	P	39	148470	-3	7.01E-09
42.	30	33553	P	30	33553	0	7.45E-09
43.	30	33562	P	30	33562	0	7.45E-09
44.	31	112629	P	31	149301	-1	1.73E-07

45.	31	112650	P	31	149322	-1	1.73E-07
46.	36	46466	P	36	121849	-3	3.51E-07
47.	36	113459	P	36	148466	-3	3.51E-07
48.	36	113480	P	36	148487	-3	3.51E-07
49.	30	8650	P	30	47909	-1	6.71E-07

Supplementary Table 12: Complement repeat in *Aquilaria hirta* chloroplast genome

No	Repeat length of the first part	Starting position of the first part	Match direction	Repeat length of the second part	Starting position of the second part	Distance of this repeat	Calculated e-value of this repeat
1.	36	33553	C	36	33556	0	1.82E-12
2.	34	33553	C	34	33558	0	2.91E-11
3.	32	33553	C	32	33560	0	4.66E-10
4.	30	33553	C	30	33562	0	7.45E-09
5.	35	33546	C	35	33557	-2	3.90E-08
6.	33	33546	C	33	33559	-2	5.53E-07
7.	31	33546	C	31	33561	-2	7.80E-06
8.	31	33546	C	31	33563	-3	2.26E-04
9.	30	30465	C	30	30467	-3	8.17E-04

Supplementary Table 13: Forward repeat in *Aquilaria malaccensis* chloroplast genome

No	Repeat length of the first part	Starting position of the first part	Match direction	Repeat length of the second part	Starting position of the second part	Distance of this repeat	Calculated e-value of this repeat
1.	163	1629	F	163	71967	-3	1.20E-81
2.	150	1630	F	150	71973	-3	1.50E-78
3.	156	1642	F	156	71980	-3	1.73E-77
4.	78	128148	F	78	128228	-3	1.93E-31
5.	78	133828	F	78	133908	-3	1.93E-31
6.	70	113389	F	70	113458	-1	1.30E-30
7.	70	148606	F	70	148675	-1	1.30E-30
8.	62	148614	F	62	148683	0	4.04E-28
9.	64	133842	F	64	133922	-1	4.85E-27
10.	62	70233	F	62	70293	-1	7.52E-26
11.	63	128168	F	63	128248	-3	1.08E-22
12.	59	112672	F	59	112693	-2	3.98E-22
13.	59	149382	F	59	149403	-2	3.98E-22
14.	57	70210	F	57	70228	-2	5.95E-21
15.	51	149390	F	51	149411	-1	2.59E-19
16.	50	70214	F	50	70292	-1	1.02E-18
17.	44	113523	F	44	113565	0	2.78E-17
18.	44	148525	F	44	148567	0	2.78E-17
19.	41	95910	F	41	95928	0	1.78E-15
20.	41	166165	F	41	166183	0	1.78E-15
21.	43	97902	F	43	164189	-1	1.43E-14
22.	38	112672	F	38	112714	-1	1.30E-11
23.	38	113533	F	38	113596	-1	1.30E-11
24.	38	148500	F	38	148563	-1	1.30E-11
25.	38	149382	F	38	149424	-1	1.30E-11
26.	41	41195	F	41	43419	-2	1.31E-11
27.	41	101941	F	41	140172	-2	1.31E-11
28.	41	121921	F	41	160152	-2	1.31E-11
29.	39	70210	F	39	70246	-2	1.90E-10
30.	36	46454	F	36	101944	-1	1.97E-10
31.	41	1526	F	41	71860	-3	5.12E-10
32.	41	1535	F	41	71869	-3	5.12E-10
33.	41	113940	F	41	113979	-3	5.12E-10
34.	41	148114	F	41	148153	-3	5.12E-10
35.	38	94603	F	38	94621	-2	7.20E-10
36.	38	167475	F	38	167493	-2	7.20E-10
37.	31	59855	F	31	59885	0	1.86E-09
38.	34	70251	F	34	70293	-1	2.97E-09
39.	34	113575	F	34	113596	-1	2.97E-09
40.	34	148504	F	34	148525	-1	2.97E-09
41.	39	113547	F	39	113610	-3	7.02E-09
42.	39	148485	F	39	148548	-3	7.02E-09
43.	30	149411	F	30	149432	0	7.46E-09
44.	36	70210	F	36	70306	-2	1.03E-08
45.	31	70293	F	31	70311	-1	1.73E-07
46.	31	112682	F	31	112724	-1	1.73E-07
47.	36	46454	F	36	140175	-3	3.51E-07

48.	36	113533	F	36	113554	-3	3.51E-07
49.	36	148544	F	36	148565	-3	3.51E-07
50.	30	46463	F	30	101953	-1	6.71E-07
51.	34	113554	F	34	113575	-3	4.71E-06

Supplementary Table 14: Reverse repeat in *Aquilaria malaccensis* chloroplast genome

No	Repeat length of the first part	Starting position of the first part	Match direction	Repeat length of the second part	Starting position of the second part	Distance of this repeat	Calculated e-value of this repeat
1.	31	33569	R	31	33569	0	1.86E-09
2.	35	33568	R	35	33568	-2	3.90E-08
3.	31	33571	R	31	33571	-2	7.80E-06

Supplementary Table 15: Palindrome repeat in *Aquilaria malaccensis* chloroplast genome

No	Repeat length of the first part	Starting position of the first part	Match direction	Repeat length of the second part	Starting position of the second part	Distance of this repeat	Calculated e-value of this repeat
1.	78	128148	P	78	133828	-3	1.93E-31
2.	78	128228	P	78	133908	-3	1.93E-31
3.	70	113389	P	70	148606	-1	1.30E-30
4.	70	113458	P	70	148675	-1	1.30E-30
5.	54	10416	P	54	10416	0	2.65E-23
6.	63	128168	P	63	133823	-3	1.08E-22
7.	63	128248	P	63	133903	-3	1.08E-22
8.	59	112672	P	59	149382	-2	3.98E-22
9.	59	112693	P	59	149403	-2	3.98E-22
10.	44	113523	P	44	148525	0	2.78E-17
11.	44	113565	P	44	148567	0	2.78E-17
12.	41	95910	P	41	166165	0	1.78E-15
13.	41	95928	P	41	166183	0	1.78E-15
14.	49	77050	P	49	77050	-3	1.35E-14
15.	43	97902	P	43	97902	-1	1.43E-14
16.	43	164189	P	43	164189	-1	1.43E-14
17.	38	112672	P	38	149382	-1	1.30E-11
18.	38	112714	P	38	149424	-1	1.30E-11
19.	38	113533	P	38	148500	-1	1.30E-11
20.	38	113596	P	38	148563	-1	1.30E-11
21.	41	101941	P	41	121921	-2	1.31E-11
22.	41	140172	P	41	160152	-2	1.31E-11
23.	36	46454	P	36	160154	-1	1.97E-10
24.	41	38603	P	41	38603	-3	5.12E-10
25.	41	113940	P	41	148114	-3	5.12E-10
26.	41	113979	P	41	148153	-3	5.12E-10
27.	38	94603	P	38	167475	-2	7.20E-10
28.	38	94621	P	38	167493	-2	7.20E-10
29.	34	113575	P	34	148504	-1	2.97E-09
30.	34	113596	P	34	148525	-1	2.97E-09
31.	39	113547	P	39	148485	-3	7.02E-09
32.	39	113610	P	39	148548	-3	7.02E-09
33.	31	112682	P	31	149379	-1	1.73E-07
34.	31	112724	P	31	149421	-1	1.73E-07
35.	36	46454	P	36	121923	-3	3.51E-07
36.	36	113533	P	36	148544	-3	3.51E-07
37.	36	113554	P	36	148565	-3	3.51E-07
38.	30	8661	P	30	47899	-1	6.71E-07
39.	30	46463	P	30	160151	-1	6.71E-07
40.	32	33569	P	32	33569	-2	2.08E-06
41.	32	33570	P	32	33570	-2	2.08E-06
42.	34	113554	P	34	148525	-3	4.71E-06
43.	34	113575	P	34	148546	-3	4.71E-06
44.	31	69260	P	31	69298	-2	7.80E-06
45.	31	112703	P	31	149379	-2	7.80E-06
46.	31	112724	P	31	149400	-2	7.80E-06
47.	30	33569	P	30	33569	-2	2.92E-05

48.	30	33572	P	30	33572	-2	2.92E-05
49.	32	4880	P	32	4918	-3	6.24E-05

Supplementary Table 16: Complement repeat in *Aquilaria malaccensis* chloroplast genome

No	Repeat length of the first part	Starting position of the first part	Match direction	Repeat length of the second part	Starting position of the second part	Distance of this repeat	Calculated e-value of this repeat
1.	32	33569	C	32	33570	-2	2.08E-06
2.	30	33569	C	30	33572	-2	2.92E-05
3.	44	37472	C	44	37512	-2	2.32E-06
4.	56	37472	C	56	37515	-2	2.31E-06

Supplementary Table 17: Forward repeat in *Aquilaria microcarpa* chloroplast genome

No	Repeat length of the first part	Starting position of the first part	Match direction	Repeat length of the second part	Starting position of the second part	Distance of this repeat	Calculated e-value of this repeat
1.	156	1642	F	156	71977	-3	1.73E-77
2.	78	128139	F	78	128219	-3	1.93E-31
3.	78	133820	F	78	133900	-3	1.93E-31
4.	70	113380	F	70	113449	-1	1.30E-30
5.	70	148598	F	70	148667	-1	1.30E-30
6.	62	148606	F	62	148675	0	4.04E-28
7.	64	133834	F	64	133914	-1	4.85E-27
8.	62	70230	F	62	70290	-1	7.52E-26
9.	63	128159	F	63	128239	-3	1.08E-22
10.	59	112663	F	59	112684	-2	3.98E-22
11.	59	149374	F	59	149395	-2	3.98E-22
12.	57	70207	F	57	70225	-2	5.94E-21
13.	51	149382	F	51	149403	-1	2.59E-19
14.	50	70211	F	50	70289	-1	1.02E-18
15.	44	113514	F	44	113556	0	2.78E-17
16.	44	148517	F	44	148559	0	2.78E-17
17.	41	95906	F	41	95924	0	1.78E-15
18.	41	166152	F	41	166170	0	1.78E-15
19.	43	97898	F	43	164176	-1	1.43E-14
20.	38	112663	F	38	112705	-1	1.30E-11
21.	38	113524	F	38	113587	-1	1.30E-11
22.	38	148492	F	38	148555	-1	1.30E-11
23.	38	149374	F	38	149416	-1	1.30E-11
24.	41	41197	F	41	43421	-2	1.31E-11
25.	41	101937	F	41	140164	-2	1.31E-11
26.	41	121912	F	41	160139	-2	1.31E-11
27.	39	70207	F	39	70243	-2	1.90E-10
28.	36	46458	F	36	101940	-1	1.97E-10
29.	41	1526	F	41	71857	-3	5.12E-10
30.	41	1535	F	41	71866	-3	5.12E-10
31.	41	113931	F	41	113970	-3	5.12E-10
32.	41	148106	F	41	148145	-3	5.12E-10
33.	38	94599	F	38	94617	-2	7.20E-10
34.	38	167462	F	38	167480	-2	7.20E-10
35.	31	59854	F	31	59884	0	1.86E-09
36.	34	70248	F	34	70290	-1	2.97E-09
37.	34	113566	F	34	113587	-1	2.97E-09
38.	34	148496	F	34	148517	-1	2.97E-09
39.	39	113538	F	39	113601	-3	7.02E-09
40.	39	148477	F	39	148540	-3	7.02E-09
41.	30	149403	F	30	149424	0	7.46E-09
42.	36	70207	F	36	70303	-2	1.03E-08
43.	31	70290	F	31	70308	-1	1.73E-07

44.	31	112673	F	31	112715	-1	1.73E-07
45.	36	46458	F	36	140167	-3	3.51E-07
46.	36	113524	F	36	113545	-3	3.51E-07
47.	36	148536	F	36	148557	-3	3.51E-07
48.	30	46467	F	30	101949	-1	6.71E-07
49.	34	113545	F	34	113566	-3	4.71E-06

Supplementary Table 18: Reverse repeat in *Aquilaria microcarpa* chloroplast genome

No	Repeat length of the first part	Starting position of the first part	Match direction	Repeat length of the second part	Starting position of the second part	Distance of this repeat	Calculated e-value of this repeat
1.	30	33570	R	30	33573	-1	6.71E-07
2.	31	33570	R	31	33570	-2	7.80E-06

Supplementary Table 19: Palindrome repeat in *Aquilaria microcarpa* chloroplast genome

No	Repeat length of the first part	Starting position of the first part	Match direction	Repeat length of the second part	Starting position of the second part	Distance of this repeat	Calculated e-value of this repeat
1.	78	128219	P	78	133900	-3	1.93E-31
2.	70	113380	P	70	148598	-1	1.30E-30
3.	70	113449	P	70	148667	-1	1.30E-30
4.	54	10412	P	54	10412	0	2.65E-23
5.	63	128159	P	63	133815	-3	1.08E-22
6.	63	128239	P	63	133895	-3	1.08E-22
7.	59	112663	P	59	149374	-2	3.98E-22
8.	59	112684	P	59	149395	-2	3.98E-22
9.	44	113514	P	44	148517	0	2.78E-17
10.	44	113556	P	44	148559	0	2.78E-17
11.	41	95906	P	41	166152	0	1.78E-15
12.	41	95924	P	41	166170	0	1.78E-15
13.	49	77047	P	49	77047	-3	1.35E-14
14.	43	97898	P	43	97898	-1	1.43E-14
15.	43	164176	P	43	164176	-1	1.43E-14
16.	38	112663	P	38	149374	-1	1.30E-11
17.	38	112705	P	38	149416	-1	1.30E-11
18.	38	113524	P	38	148492	-1	1.30E-11
19.	38	113587	P	38	148555	-1	1.30E-11
20.	41	101937	P	41	121912	-2	1.31E-11
21.	41	140164	P	41	160139	-2	1.31E-11
22.	36	46458	P	36	160141	-1	1.97E-10
23.	41	38601	P	41	38601	-3	5.12E-10
24.	41	113931	P	41	148106	-3	5.12E-10
25.	41	113970	P	41	148145	-3	5.12E-10
26.	38	94599	P	38	167462	-2	7.20E-10
27.	38	94617	P	38	167480	-2	7.20E-10
28.	34	113566	P	34	148496	-1	2.97E-09
29.	34	113587	P	34	148517	-1	2.97E-09
30.	39	113538	P	39	148477	-3	7.02E-09
31.	39	113601	P	39	148540	-3	7.02E-09
32.	31	112673	P	31	149371	-1	1.73E-07
33.	31	112715	P	31	149413	-1	1.73E-07
34.	36	46458	P	36	121914	-3	3.51E-07
35.	36	113524	P	36	148536	-3	3.51E-07
36.	36	113545	P	36	148557	-3	3.51E-07
37.	30	8656	P	30	47903	-1	6.71E-07
38.	30	46467	P	30	160138	-1	6.71E-07
39.	34	113545	P	34	148517	-3	4.71E-06
40.	34	113566	P	34	148538	-3	4.71E-06
41.	31	69257	P	31	69295	-2	7.80E-06
42.	31	112694	P	31	149371	-2	7.80E-06
43.	31	112715	P	31	149392	-2	7.80E-06

44.	32	4881	P	32	4919	-3	6.24E-05
45.	32	118507	P	32	118553	-3	6.24E-05
46.	32	126697	P	32	135320	-3	6.24E-05
47.	32	126765	P	32	135388	-3	6.24E-05
48.	32	143532	P	32	143578	-3	6.24E-05

Supplementary Table 20: Complement repeat in *Aquilaria microcarpa* chloroplast genome

No	Repeat length of the first part	Starting position of the first part	Match direction	Repeat length of the second part	Starting position of the second part	Distance of this repeat	Calculated e-value of this repeat
1.	30	30480	C	30	30482	-3	8.17E-04

Supplementary Table 21: Forward repeat in *Aquilaria rostrata* chloroplast genome

No	Repeat length of the first part	Starting position of the first part	Match direction	Repeat length of the second part	Starting position of the second part	Distance of this repeat	Calculated e-value of this repeat
1.	152	1629	F	152	71915	-2	2.72E-77
2.	150	1642	F	150	71928	-3	6.27E-74
3.	137	1661	F	137	71947	-3	3.20E-66
4.	78	128108	F	78	128188	-3	1.93E-31
5.	78	133682	F	78	133762	-3	1.93E-31
6.	70	113349	F	70	113418	-1	1.29E-30
7.	70	148460	F	70	148529	-1	1.29E-30
8.	62	148468	F	62	148537	0	4.04E-28
9.	64	133696	F	64	133776	-1	4.84E-27
10.	62	70187	F	62	70247	-2	6.87E-24
11.	62	112632	F	62	112653	-2	6.87E-24
12.	62	149233	F	62	149254	-2	6.87E-24
13.	63	128128	F	63	128208	-3	1.08E-22
14.	57	70164	F	57	70182	-2	5.94E-21
15.	50	70168	F	50	70246	0	6.77E-21
16.	51	149244	F	51	149265	-1	2.59E-19
17.	44	113483	F	44	113525	0	2.77E-17
18.	44	148379	F	44	148421	0	2.77E-17
19.	41	95863	F	41	95881	0	1.77E-15
20.	41	166026	F	41	166044	0	1.77E-15
21.	41	70208	F	41	70268	-1	2.18E-13
22.	41	112632	F	41	112674	-1	2.18E-13
23.	41	149233	F	41	149275	-1	2.18E-13
24.	38	113493	F	38	113556	-1	1.29E-11
25.	38	148354	F	38	148417	-1	1.29E-11
26.	41	41152	F	41	43376	-2	1.31E-11
27.	41	101901	F	41	140026	-2	1.31E-11
28.	41	121881	F	41	160006	-2	1.31E-11
29.	36	46416	F	36	101904	-1	1.96E-10
30.	41	1526	F	41	71808	-3	5.11E-10
31.	41	1535	F	41	71817	-3	5.11E-10
32.	41	113900	F	41	113939	-3	5.11E-10
33.	41	147968	F	41	148007	-3	5.11E-10
34.	38	94556	F	38	94574	-2	7.19E-10
35.	38	167336	F	38	167354	-2	7.19E-10
36.	31	59810	F	31	59840	0	1.86E-09
37.	31	70190	F	31	70208	0	1.86E-09
38.	31	112642	F	31	112684	0	1.86E-09
39.	34	113535	F	34	113556	-1	2.97E-09
40.	34	148358	F	34	148379	-1	2.97E-09
41.	39	70164	F	39	70200	-3	7.01E-09
42.	39	113507	F	39	113570	-3	7.01E-09
43.	39	148339	F	39	148402	-3	7.01E-09
44.	30	149265	F	30	149286	0	7.44E-09

45.	36	70164	F	36	70260	-2	1.03E-08
46.	31	70208	F	31	70250	-1	1.73E-07
47.	31	70247	F	31	70265	-1	1.73E-07
48.	31	112663	F	31	112684	-1	1.73E-07
49.	36	46416	F	36	140029	-3	3.50E-07
50.	36	113493	F	36	113514	-3	3.50E-07

Supplementary Table 22: Reverse repeat in *Aquilaria rostrata* chloroplast genome

No	Repeat length of the first part	Starting position of the first part	Match direction	Repeat length of the second part	Starting position of the second part	Distance of this repeat	Calculated e-value of this repeat
1.	30	33528	R	30	33531	-1	6.70E-07
2.	31	33528	R	31	33528	-2	7.79E-06

Supplementary Table 23: Palindrome repeat in *Aquilaria rostrata* chloroplast genome

No	Repeat length of the first part	Starting position of the first part	Match direction	Repeat length of the second part	Starting position of the second part	Distance of this repeat	Calculated e-value of this repeat
1.	78	128188	P	78	133762	-3	1.93E-31
2.	70	113349	P	70	148460	-1	1.29E-30
3.	70	113418	P	70	148529	-1	1.29E-30
4.	62	112632	P	62	149233	-2	6.87E-24
5.	62	112653	P	62	149254	-2	6.87E-24
6.	63	128128	P	63	133677	-3	1.08E-22
7.	63	128208	P	63	133757	-3	1.08E-22
8.	54	10375	P	54	10375	-2	3.41E-19
9.	44	30427	P	44	30427	0	2.77E-17
10.	44	113483	P	44	148379	0	2.77E-17
11.	44	113525	P	44	148421	0	2.77E-17
12.	41	95863	P	41	166026	0	1.77E-15
13.	41	95881	P	41	166044	0	1.77E-15
14.	49	76998	P	49	76998	-3	1.35E-14
15.	41	112632	P	41	149233	-1	2.18E-13
16.	41	112674	P	41	149275	-1	2.18E-13
17.	44	49006	P	44	49006	-2	2.36E-13
18.	38	113493	P	38	148354	-1	1.29E-11
19.	38	113556	P	38	148417	-1	1.29E-11
20.	41	101901	P	41	121881	-2	1.31E-11
21.	41	140026	P	41	160006	-2	1.31E-11
22.	43	38561	P	43	38561	-3	3.70E-11
23.	36	46416	P	36	160008	-1	1.96E-10
24.	41	113900	P	41	147968	-3	5.11E-10
25.	41	113939	P	41	148007	-3	5.11E-10
26.	38	94556	P	38	167336	-2	7.19E-10
27.	38	94574	P	38	167354	-2	7.19E-10
28.	31	112642	P	31	149233	0	1.86E-09
29.	31	112684	P	31	149275	0	1.86E-09
30.	34	113535	P	34	148358	-1	2.97E-09
31.	34	113556	P	34	148379	-1	2.97E-09
32.	39	113507	P	39	148339	-3	7.01E-09
33.	39	113570	P	39	148402	-3	7.01E-09
34.	31	112663	P	31	149233	-1	1.73E-07
35.	31	112684	P	31	149254	-1	1.73E-07
36.	36	46416	P	36	121883	-3	3.50E-07
37.	36	113493	P	36	148398	-3	3.50E-07
38.	36	113514	P	36	148419	-3	3.50E-07
39.	30	8629	P	30	47861	-1	6.70E-07
40.	30	46425	P	30	160005	-1	6.70E-07
41.	34	113514	P	34	148379	-3	4.70E-06
42.	34	113535	P	34	148400	-3	4.70E-06
43.	31	69214	P	31	69252	-2	7.79E-06
44.	30	14110	P	30	14110	-2	2.91E-05

45.	32	4878	P	32	4916	-3	6.23E-05
46.	32	118476	P	32	118522	-3	6.23E-05
47.	32	126666	P	32	135182	-3	6.23E-05
48.	32	126734	P	32	135250	-3	6.23E-05

Supplementary Table 24: Forward repeat in *Aquilaria sinensis* chloroplast genome

No	Repeat length of the first part	Starting position of the first part	Match direction	Repeat length of the second part	Starting position of the second part	Distance of this repeat	Calculated e-value of this repeat
1.	163	1633	F	163	72016	-3	1.20E-81
2.	156	1646	F	156	72029	-3	1.73E-77
3.	78	128207	F	78	128287	-3	1.93E-31
4.	78	133897	F	78	133977	-3	1.93E-31
5.	70	113448	F	70	113517	-1	1.30E-30
6.	70	148675	F	70	148744	-1	1.30E-30
7.	62	148683	F	62	148752	0	4.05E-28
8.	64	133911	F	64	133991	-1	4.85E-27
9.	67	70258	F	67	70336	-2	7.86E-27
10.	62	112731	F	62	112752	-2	6.89E-24
11.	62	149448	F	62	149469	-2	6.89E-24
12.	63	128227	F	63	128307	-3	1.08E-22
13.	53	70262	F	53	70322	-1	1.69E-20
14.	51	149459	F	51	149480	-1	2.60E-19
15.	49	70323	F	49	70341	-1	3.99E-18
16.	44	113582	F	44	113624	0	2.78E-17
17.	44	148594	F	44	148636	0	2.78E-17
18.	41	95963	F	41	95981	0	1.78E-15
19.	41	166240	F	41	166258	0	1.78E-15
20.	41	112731	F	41	112773	-1	2.19E-13
21.	41	149448	F	41	149490	-1	2.19E-13
22.	38	113592	F	38	113655	-1	1.30E-11
23.	38	148569	F	38	148632	-1	1.30E-11
24.	41	41214	F	41	43438	-2	1.31E-11
25.	41	102001	F	41	140241	-2	1.31E-11
26.	41	121980	F	41	160220	-2	1.31E-11
27.	39	70258	F	39	70276	-2	1.90E-10
28.	36	46477	F	36	102004	-1	1.97E-10
29.	32	31863	F	32	31880	0	4.66E-10
30.	41	1530	F	41	71909	-3	5.12E-10
31.	41	1539	F	41	71918	-3	5.12E-10
32.	41	113999	F	41	114038	-3	5.12E-10
33.	41	148183	F	41	148222	-3	5.12E-10
34.	38	94656	F	38	94674	-2	7.20E-10
35.	38	167550	F	38	167568	-2	7.20E-10
36.	31	59899	F	31	59929	0	1.87E-09
37.	31	112741	F	31	112783	0	1.87E-09
38.	34	70281	F	34	70323	-1	2.97E-09
39.	34	113634	F	34	113655	-1	2.97E-09
40.	34	148573	F	34	148594	-1	2.97E-09
41.	39	113606	F	39	113669	-3	7.02E-09
42.	39	148554	F	39	148617	-3	7.02E-09
43.	30	149480	F	30	149501	0	7.46E-09
44.	36	70258	F	36	70354	-2	1.03E-08

45.	31	70323	F	31	70359	-1	1.74E-07
46.	31	112762	F	31	112783	-1	1.74E-07
47.	36	46477	F	36	140244	-3	3.51E-07
48.	36	113592	F	36	113613	-3	3.51E-07
49.	36	148613	F	36	148634	-3	3.51E-07
50.	30	46486	F	30	102013	-1	6.72E-07

Supplementary Table 25: Reverse repeat in *Aquilaria sinensis* chloroplast genome

No	Repeat length of the first part	Starting position of the first part	Match direction	Repeat length of the second part	Starting position of the second part	Distance of this repeat	Calculated e-value of this repeat
1.	30	33581	R	30	33584	-1	6.72E-07
2.	31	33581	R	31	33581	-2	7.81E-06

Supplementary Table 26: Palindrome repeat in *Aquilaria sinensis* chloroplast genome

No	Repeat length of the first part	Starting position of the first part	Match direction	Repeat length of the second part	Starting position of the second part	Distance of this repeat	Calculated e-value of this repeat
1.	70	113448	P	70	148675	-1	1.30E-30
2.	70	113517	P	70	148744	-1	1.30E-30
3.	62	112731	P	62	149448	-2	6.89E-24
4.	62	112752	P	62	149469	-2	6.89E-24
5.	54	10422	P	54	10422	0	2.65E-23
6.	63	128227	P	63	133892	-3	1.08E-22
7.	63	128307	P	63	133972	-3	1.08E-22
8.	44	113582	P	44	148594	0	2.78E-17
9.	44	113624	P	44	148636	0	2.78E-17
10.	41	95963	P	41	166240	0	1.78E-15
11.	41	95981	P	41	166258	0	1.78E-15
12.	49	77099	P	49	77099	-3	1.35E-14
13.	41	112731	P	41	149448	-1	2.19E-13
14.	41	112773	P	41	149490	-1	2.19E-13
15.	38	113592	P	38	148569	-1	1.30E-11
16.	38	113655	P	38	148632	-1	1.30E-11
17.	41	102001	P	41	121980	-2	1.31E-11
18.	41	140241	P	41	160220	-2	1.31E-11
19.	36	46477	P	36	160222	-1	1.97E-10
20.	41	38619	P	41	38619	-3	5.12E-10
21.	41	113999	P	41	148183	-3	5.12E-10
22.	41	114038	P	41	148222	-3	5.12E-10
23.	38	94656	P	38	167550	-2	7.20E-10
24.	38	94674	P	38	167568	-2	7.20E-10
25.	31	112741	P	31	149448	0	1.87E-09
26.	31	112783	P	31	149490	0	1.87E-09
27.	34	113634	P	34	148573	-1	2.97E-09
28.	34	113655	P	34	148594	-1	2.97E-09
29.	39	113606	P	39	148554	-3	7.02E-09
30.	39	113669	P	39	148617	-3	7.02E-09
31.	31	69306	P	31	69348	-1	1.74E-07
32.	31	112762	P	31	149448	-1	1.74E-07
33.	31	112783	P	31	149469	-1	1.74E-07
34.	36	46477	P	36	121982	-3	3.51E-07
35.	36	113592	P	36	148613	-3	3.51E-07
36.	36	113613	P	36	148634	-3	3.51E-07
37.	30	8668	P	30	47920	-1	6.72E-07
38.	30	46486	P	30	160219	-1	6.72E-07
39.	34	4884	P	34	4922	-3	4.71E-06
40.	34	113613	P	34	148594	-3	4.71E-06
41.	34	113634	P	34	148615	-3	4.71E-06
42.	32	118575	P	32	118621	-3	6.25E-05
43.	32	126765	P	32	135397	-3	6.25E-05

44.	32	126833	P	32	135465	-3	6.25E-05
45.	32	143609	P	32	143655	-3	6.25E-05
46.	31	95955	P	31	166240	-3	2.26E-04
47.	31	95991	P	31	166276	-3	2.26E-04

Supplementary Table 27: Complement repeat in *Aquilaria sinensis* chloroplast genome

No	Repeat length of the first part	Starting position of the first part	Match direction	Repeat length of the second part	Starting position of the second part	Distance of this repeat	Calculated e-value of this repeat
1.	30	30473	C	30	30475	-3	8.18E-04

Supplementary Table 28: Forward repeat in *Aquilaria subintegra* chloroplast genome

No	Repeat length of the first part	Starting position of the first part	Match direction	Repeat length of the second part	Starting position of the second part	Distance of this repeat	Calculated e-value of this repeat
1.	150	1642	F	150	71956	-3	6.28E-74
2.	137	1661	F	137	71975	-3	3.21E-66
3.	78	128132	F	78	128212	-3	1.93E-31
4.	78	133817	F	78	133897	-3	1.93E-31
5.	70	113373	F	70	113442	-1	1.30E-30
6.	70	148595	F	70	148664	-1	1.30E-30
7.	62	148603	F	62	148672	0	4.04E-28
8.	64	133831	F	64	133911	-1	4.85E-27
9.	63	70207	F	63	70267	-1	1.91E-26
10.	62	112656	F	62	112677	-2	6.88E-24
11.	62	149368	F	62	149389	-2	6.88E-24
12.	63	128152	F	63	128232	-3	1.08E-22
13.	51	149379	F	51	149400	-1	2.59E-19
14.	44	113507	F	44	113549	0	2.78E-17
15.	44	148514	F	44	148556	0	2.78E-17
16.	41	95887	F	41	95905	0	1.78E-15
17.	41	166161	F	41	166179	0	1.78E-15
18.	41	112656	F	41	112698	-1	2.19E-13
19.	41	149368	F	41	149410	-1	2.19E-13
20.	38	113517	F	38	113580	-1	1.30E-11
21.	38	148489	F	38	148552	-1	1.30E-11
22.	41	41179	F	41	43403	-2	1.31E-11
23.	41	101925	F	41	140161	-2	1.31E-11
24.	41	121905	F	41	160141	-2	1.31E-11
25.	39	70203	F	39	70221	-2	1.90E-10
26.	36	46444	F	36	101928	-1	1.97E-10
27.	41	1526	F	41	71836	-3	5.12E-10
28.	41	1535	F	41	71845	-3	5.12E-10
29.	41	113924	F	41	113963	-3	5.12E-10
30.	41	148103	F	41	148142	-3	5.12E-10
31.	38	94580	F	38	94598	-2	7.20E-10
32.	38	167471	F	38	167489	-2	7.20E-10
33.	31	59847	F	31	59877	0	1.86E-09
34.	31	112666	F	31	112708	0	1.86E-09
35.	34	70226	F	34	70268	-1	2.97E-09
36.	34	113559	F	34	113580	-1	2.97E-09
37.	34	148493	F	34	148514	-1	2.97E-09
38.	39	113531	F	39	113594	-3	7.02E-09
39.	39	148474	F	39	148537	-3	7.02E-09
40.	30	149400	F	30	149421	0	7.46E-09
41.	36	70203	F	36	70281	-2	1.03E-08
42.	31	70268	F	31	70286	-1	1.73E-07
43.	31	112687	F	31	112708	-1	1.73E-07
44.	36	46444	F	36	140164	-3	3.51E-07

45.	36	113517	F	36	113538	-3	3.51E-07
46.	36	148533	F	36	148554	-3	3.51E-07
47.	30	46453	F	30	101937	-1	6.71E-07
48.	34	113538	F	34	113559	-3	4.71E-06

Supplementary Table 29: Reverse repeat in *Aquilaria subintegra* chloroplast genome

No	Repeat length of the first part	Starting position of the first part	Match direction	Repeat length of the second part	Starting position of the second part	Distance of this repeat	Calculated e-value of this repeat
1.	30	33549	R	30	33552	-1	6.71E-07
2.	31	33549	R	31	33549	-2	7.80E-06

Supplementary Table 30: Palindrome repeat in *Aquilaria subintegra* chloroplast genome

No	Repeat length of the first part	Starting position of the first part	Match direction	Repeat length of the second part	Starting position of the second part	Distance of this repeat	Calculated e-value of this repeat
1.	70	113373	P	70	148595	-1	1.30E-30
2.	70	113442	P	70	148664	-1	1.30E-30
3.	62	112656	P	62	149368	-2	6.88E-24
4.	62	112677	P	62	149389	-2	6.88E-24
5.	54	10403	P	54	10403	0	2.65E-23
6.	63	128152	P	63	133812	-3	1.08E-22
7.	63	128232	P	63	133892	-3	1.08E-22
8.	44	113507	P	44	148514	0	2.78E-17
9.	44	113549	P	44	148556	0	2.78E-17
10.	41	95887	P	41	166161	0	1.78E-15
11.	41	95905	P	41	166179	0	1.78E-15
12.	49	77026	P	49	77026	-3	1.35E-14
13.	41	112656	P	41	149368	-1	2.19E-13
14.	41	112698	P	41	149410	-1	2.19E-13
15.	38	113517	P	38	148489	-1	1.30E-11
16.	38	113580	P	38	148552	-1	1.30E-11
17.	41	101925	P	41	121905	-2	1.31E-11
18.	41	140161	P	41	160141	-2	1.31E-11
19.	36	46444	P	36	160143	-1	1.97E-10
20.	41	38583	P	41	38583	-3	5.12E-10
21.	41	113924	P	41	148103	-3	5.12E-10
22.	41	113963	P	41	148142	-3	5.12E-10
23.	38	94580	P	38	167471	-2	7.20E-10
24.	38	94598	P	38	167489	-2	7.20E-10
25.	31	112666	P	31	149368	0	1.86E-09
26.	31	112708	P	31	149410	0	1.86E-09
27.	34	113559	P	34	148493	-1	2.97E-09
28.	34	113580	P	34	148514	-1	2.97E-09
29.	39	113531	P	39	148474	-3	7.02E-09
30.	39	113594	P	39	148537	-3	7.02E-09
31.	31	112687	P	31	149368	-1	1.73E-07
32.	31	112708	P	31	149389	-1	1.73E-07
33.	36	46444	P	36	121907	-3	3.51E-07
34.	36	113517	P	36	148533	-3	3.51E-07
35.	36	113538	P	36	148554	-3	3.51E-07
36.	30	8650	P	30	47887	-1	6.71E-07
37.	30	46453	P	30	160140	-1	6.71E-07
38.	34	113538	P	34	148514	-3	4.71E-06
39.	34	113559	P	34	148535	-3	4.71E-06
40.	31	69253	P	31	69291	-2	7.80E-06
41.	32	118500	P	32	118546	-3	6.24E-05
42.	32	126690	P	32	135317	-3	6.24E-05
43.	32	126758	P	32	135385	-3	6.24E-05

44.	32	143529	P	32	143575	-3	6.24E-05
45.	31	95879	P	31	166161	-3	2.26E-04
46.	31	95915	P	31	166197	-3	2.26E-04
47.	31	96527	P	31	96527	-3	2.26E-04

Supplementary Table 31: Complement repeat in *Aquilaria subintegra* chloroplast genome

No	Repeat length of the first part	Starting position of the first part	Match direction	Repeat length of the second part	Starting position of the second part	Distance of this repeat	Calculated e-value of this repeat
1.	30	30460	C	30	30462	-3	8.17E-04
